# Sacubitril/valsartan in Heart Failure and Beyond—From Molecular Mechanisms to Clinical Relevance

**DOI:** 10.31083/j.rcm2307238

**Published:** 2022-06-24

**Authors:** Maja Nikolic, Ivan Srejovic, Jovana Joksimovic Jovic, Jasmina Sretenovic, Jovana Jeremic, Ivan Cekerevac, Stefan Simovic, Danijela Djokovic, Nemanja Muric, Vladislava Stojic, Stefani Bolevich, Sergey Bolevich, Vladimir Jakovljevic

**Affiliations:** ^1^Department of Physiology, Faculty of Medical Sciences, University of Kragujevac, 34000 Kragujevac, Serbia; ^2^Department of Pharmacology, Faculty of Medical Sciences, University of Kragujevac, 34000 Kragujevac, Serbia; ^3^Department of Internal Medicine, Faculty of Medical Sciences, University of Kragujevac, 34000 Kragujevac, Serbia; ^4^Clinic of Pulmology, University Clinical Center Kragujevac, 34000 Kragujevac, Serbia; ^5^Clinic of Cardiology, University Clinical Center Kragujevac, 34000 Kragujevac, Serbia; ^6^Department of Psychiatry, Faculty of Medical Sciences, University of Kragujevac, 34000 Kragujevac, Serbia; ^7^Clinic of Psychiatry, University Clinical Center Kragujevac, 34000 Kragujevac, Serbia; ^8^Department of Medical Statistics and Informatics, Faculty of Medical Sciences, University of Kragujevac, 34000 Kragujevac, Serbia; ^9^Department of Pathophysiology, 1st Moscow State Medical University IM Sechenov, 119991 Moscow, Russia; ^10^Department of Human Pathology, 1st Moscow State Medical University IM Sechenov, 119991 Moscow, Russia

**Keywords:** sacubitril/valsartan, heart failure, cardiovascular diseases, cardioprotective mechanisms

## Abstract

As the ultimate pathophysiological event, heart failure (HF) may arise from various cardiovascular (CV) conditions, including sustained pressure/volume overload of the left ventricle, myocardial infarction or ischemia, and cardiomyopathies. Sacubitril/valsartan (S/V; formerly termed as LCZ696), a first-in-class angiotensin receptor/neprilysin inhibitor, brought a significant shift in the management of HF with reduced ejection fraction by modulating both renin-angiotensin-aldosterone system (angiotensin II type I receptor blockage by valsartan) and natriuretic peptide system (neprilysin inhibition by sacubitril) pathways. Besides, the efficacy of S/V has been also investigated in the setting of other CV pathologies which are during their pathophysiological course and progression deeply interrelated with HF. However, its mechanism of action is not entirely clarified, suggesting other off-target benefits contributing to its cardioprotection. In this review article our goal was to highlight up-to-date clinical and experimental evidence on S/V cardioprotective effects, as well as most discussed molecular mechanisms achieved by this dual-acting compound. Although S/V was extensively investigated in HF patients, additional large studies are needed to elucidate its effects in the setting of other CV conditions. Furthermore, with its antiinflamatory potential, this agent should be investigated in animal models of inflammatory heart diseases, such as myocarditis, while it may possibly improve cardiac dysfunction as well as inflammatory response in this pathophysiological setting. Also, discovering other signalling pathways affected by S/V should be of particular interest for basic researches, while it can provide additional understanding of its cardioprotective mechanisms.

## 1. Introduction

Cardiovascular diseases (CVDs) remain global mortality contributors with nearly doubled prevalence in the last three decades [1]. As the ultimate pathophysiological event, heart failure (HF) may arise from various cardiovascular (CV) conditions, including sustained pressure/volume overload of the left ventricle (LV), myocardial infarction (MI) or ischemia, and cardiomyopathies [2]. The devastating prevalence of HF, which affects more than 64 million people worldwide [3], underscores the great importance of implementing novel therapeutic strategies in this population of patients.

According to the European Society of Cardiology (ESC), currently accepted HF categorization, based on LV ejection fraction (EF) (LVEF), divides HF patients into following subgroups: HF with reduced EF (HFrEF, EF ≤40%), mildly reduced EF (HFmEF, EF 41–49%), and preserved EF (HFpEF, EF ≥50%) [4]. Over the past few decades, identifying the main neurohormonal mechanisms involved in the occurrence and progression of HF was a crucial step forward in developing broad therapeutic artillery for its management. Pharmacological targeting of the neuroendocrine dysregulation of the sympathetic nervous system (SNS) by beta-blockers and the renin-angiotensin-aldosterone system (RAAS) by angiotensin-converting enzyme (ACE) inhibitors (ACEi), angiotensin receptor blockers (ARBs), and mineralocorticoid receptor antagonists (MRAs) represents a cornerstone of a modern approach for the management of HFrEF [5]. However, the expected benefit from the guideline-approved therapies has not yet been reached, considering the 5-year survival rates in patients with HF are as poor as in patients diagnosed with cancer [6]. Furthermore, well-established therapeutics for the management of HFrEF have not proven effective in HFpEF patients, which constitutes nearly 50% of the HF population [7].

The knowledge of the natriuretic peptide system (NPS) paved the way for continuous research into therapeutic options for HF. The possibility of augmenting NPS by neprilysin (NEP) inhibition, and therefore amplifying the desired physiological actions of natriuretic peptides (NPs) has gained considerable scientific interest. Sacubitril/valsartan (S/V; formerly termed as LCZ696), a first-in-class angiotensin receptor/neprilysin inhibitor (ARNI), brought a significant shift in the management of patients with HFrEF by modulating both NPS (NEP inhibition by sacubitril) and RAAS (angiotensin II type I receptor blockage by valsartan) pathways. The pivotal evidence on its overwhelming benefits arrived in 2014 from a landmark, the PARADIGM-HF (Prospective Comparison of ARNI with angiotensin-converting enzyme inhibitor (ACEI) to Determine Impact on Global Mortality and Morbidity in Heart Failure) trial [8], which subsequently changed the recommendations for HFrEF therapeutic approach [5, 9].

It is worth mentioning that sodium-glucose co-transporter 2 inhibitors (SGLT2i) have recently also strengthened the therapeutic artillery for the management of HFrEF [4, 10]. Although initially developed as antidiabetics, these agents (empagliflozin and dapagliflozin) were proved effective in reducing CV death and HF hospitalization in HFrEF patients, irrespectively of their diabetes status [11, 12]. The landmark trial DAPA-HF (Dapagliflozin and Prevention of Adverse Outcomes in Heart Failure) demonstrated that in comparison to the placebo, dapagliflozin significantly reduced the incidence of CV death and HF worsening in HFrEF patients regardless of the presence of diabetes [11]. Similar results arrived from the EMPEROR-REDUCED (Empagliflozin Outcome Trial in Patients with Chronic Heart Failure and a Reduced Ejection Fraction) trial, where empagliflozin proved its superiority against placebo in HFrEF patients by reducing the relative risk of CV mortality and HF hospitalization, irrespectively of their diabetes status [12]. Furthermore, SGLT2i were equally effective in HFrEF patients with or without S/V treatment, suggesting that the use of both agents could achieve the most prominent cardioprotective effect in this population of patients [13, 14].

In recent years, the efficacy of S/V has been also investigated in the setting of other CV pathologies which are during their pathophysiological course and progression deeply interrelated with HF. However, its mechanism of action is still not entirely clarified, suggesting other off-target effects contributing to its cardioprotection.

Herein, we aimed to highlight up-to date clinical and experimental evidence on S/V cardioprotective effects, as well as the most discussed molecular mechanisms.

## 2. Natriuretic Peptide System in Brief

NPs are a family of structurally similar but genetically distinct bioactive peptides involving atrial natriuretic peptide (ANP), B-type (or brain) natriuretic peptide (BNP), and C-type natriuretic peptide (CNP) [15]. A surge of scientific interest for NPs began in 1981 after Bold and colleagues [16] observed increased diuresis (>10-fold) and natriuresis (>30-fold) followed by a reduction in blood pressure (BP) in rats intravenously administered with a rat atrial extract. The first member of this family, ANP, has been isolated from animal and human heart atria [17, 18]. Subsequently, BNP was primarily isolated from extracts of porcine brain tissue [19]. However, further investigations reported its highest concentration in cardiac ventricles of HF patients [20, 21]. Similarly, CNP was also firstly identified in the porcine brain extracts [22], and it is majorly secreted from the brain, chondrocytes, and endothelial cells [23]. Both ANP and BNP are secreted from cardiac atria and ventricles, respectively, as a response to myocardial stretching and mainly regulate fluid volume and BP homeostasis [24]. All three NPs are synthesized as pre-prohormones, and their actions are related to interaction with specific receptors. To date, three natriuretic peptide receptors (NPRs) have been identified as a part of NPS. NPR-A and NPR-B are transmembrane guanylyl cyclase enzymes responsible for catalyzation of second messenger, cyclic guanosine monophosphate (cGMP), which in turn mediates various signalling cascades in the target organs, inducing vasodilatation, natriuresis and diuresis, inhibition of RAAS, endothelin and vasopressin, and lipid mobilization [25, 26]. Both ANP and BNP majorly binds to NPR-A, CNP binds to NPR-B, whereas all three can bind to NPR-C [27], which acts as a clearance receptor of NPs [28].

Two known pathways can accomplish NPs breakdown: (1) NRP-C-mediated internalization and lysosomal degradation [28, 29], (2) enzymatic degradation performed by NEP, a zinc-dependent metalloprotease that is widely distributed in various tissues, such as endothelial and epithelial tissue, smooth muscle cells, cardiac myocytes, adipocytes, and pancreatic islets [30]. It is considered a principal enzyme for the degradation of numerous vasoactive peptides with different physiological roles (with vasodilatator or vasoconstrictive effects) in the CV system. NEP exhibits the greatest affinity for ANP, CNP, angiotensin I and II, while the lowest for BNP, endothelin-1, and bradykinin [23]. Unlike NRP-C, NEP metabolism has a minor contribution in NPs clearance under normal conditions, whereas, in pathological states with an increased level of circulating NPs, it is the dominant mode of NPs breakdown. On the contrary, NPR-C may become saturated [31].

Except in maintaining BP homeostasis, NPs also exhibit a diverse myriad of physiological effects, including antifibrotic, antihypertrophic, anti-inflammatory, and lusitropic effects, as well as sympathoinhibition and RAAS suppression [32, 33]. In most of their physiological aspects, NPs act antagonistically to RAAS. In HF, NPs also has a significant diagnostic role. In particular, BNP and its inactive terminal fragment, N-terminal pro b-type natriuretic peptide (NT-proBNP) plasma levels rise in response to increased ventricular stress and are considered a gold standard for HF diagnosis, prediction of its severity and prognosis [5, 9]. However, despite the significant elevation of NPs in congestive HF, its protective effects diminish as the disease progress, with a dominance of RAAS and SNS in disease deterioration [34]. In his recently published paper, Diez comprehensively described a variety of potential mechanisms considered to decrease the beneficial effects of NPs in the state of chronic HF [34]. However, we will not discuss it here, while it was not the focus of our present review.

Given the adopted knowledge on favourable actions of NPs, the goal of numerous studies that followed was to develop an appropriate therapeutic weapon that would enhance its effects and facilitate HF management.

## 3. Long Road to ARNI

An urgent need for manufacturing novel agents for HF treatment led to an explosion of investigations in recent years. Initial studies related to maximizing the NPs physiological actions aimed to examine the efficacy of exogenous NPs administration in HFrEF patients, however, the results were not promising. Nesiritide, a recombinant human BNP, beneficially influenced hemodynamics in the setting of acute decompensation of chronic HF [35], however it failed to reduce mortality and HF rehospitalization in comparison to placebo [36]. On the other hand, carperitide, a recombinant human ANP, is widely used in patients with acute HF in Japan [37], although no robust evidence proved its efficacy in improving clinical end-points [38]. On contrary, one recent cohort even reported that the use of carperitide was associated with worse outcomes in patients with acute HF than those assigned to nitrates [39]. Therefore, another strategy for endogenous NP reinforcement rushed into scientific focus concerning inhibition of NEP-mediated NPs’ breakdown.

In 1980, Roques and colleagues [40] reported the earliest preclinical findings on NEP inhibition effects. In healthy individuals, administration of NEP inhibitors as monotherapy improved natriuresis and diuresis and increase plasma ANP levels [41, 42]. However, despite an increase of ANP due to chronic use of candoxatril, an oral NEP inhibitor, sustained lowering of BP in hypertensive patients has not been achieved, so further research on its usage was discontinued [43]. As discussed earlier, there are various NEP substrates, including those with vasoconstrictive effects (angiotensin or endothelin). Therefore, suppression of its physiological actions not only increases the plasma levels of NPs, but vasoconstrictor levels too [6], leading to annulation of its preferred effects. Nevertheless, to overcome the shortcomings of lone NEP inhibition, manufacturing a dual-acting compound that would simultaneously activate NPS and inhibit RAAS seemed like a convenient solution.

After exceptional results from extensive clinical studies, CONSENSUS and SOLVD-Treatment trials [44, 45], ACEi have strengthened their place in the treatment of HFrEF for more than 30 years. Therefore, these agents were first tested in synergy with NEP inhibitors. However, in the OVERTURE trial, omapratilat, a first combined ACE and NEP inhibitor, was not superior over enalapril in reduction of the primary clinical event in patients with chronic HF [46]. Furthermore, this drug was associated with an unacceptable risk of angioedema [46], which was attributed to its dual mechanism of action; inhibiting both ACE and NEP, which are responsible for the breakdown of bradykinin, led to its excessive accumulation and consequent angioedema [47]. From this moment, the most promising drug class for combination with NEP inhibitor became ARBs.

A first-in-class ARNI is comprised of two molecular moieties in a 1:1 M ratio, sacubitril, which is rapidly metabolized after oral ingestion into sacubitrilat, NEP inhibitor, and valsartan, a well-known ARB with established efficacy in treating CVDs [48]. S/V primarily targets two neurohormonal pathways critical for the pathophysiology of HF and thus increase plasma cGMP concentration as a consequence of enhanced NPS in one side and plasma renin and angiotensin II mediated by ARB on the other [49, 50].

## 4. S/V in HF and Beyond 

**Fig. 1.**
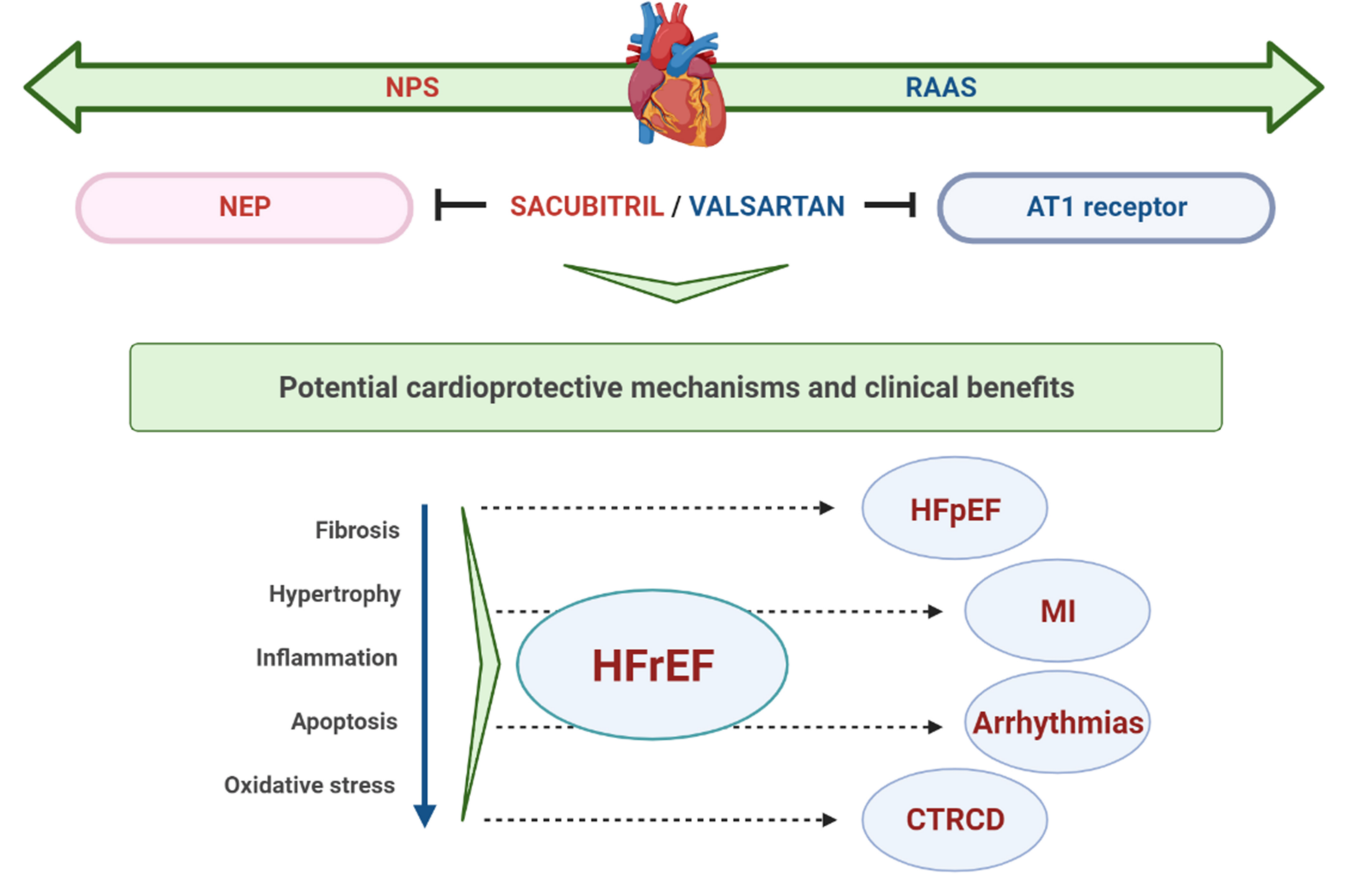
**Schematic representation of potential cardioprotective mechanisms and clinical benefits of S/V**. NPS, natriuretic peptide system; RAAS, renin-angiotensin-aldosterone system; NEP, neprylisin; AT1, angiotensin II type I; HFrEF, heart failure with reduced ejection fraction; HFpEF, heart failure with preserved ejection fraction; MI, myocardial infarction; CTRCD, cancer therapy-related cardiac dysfunction.

With increasing evidence on its protective effects in HFrEF [8], S/V has also been evaluated in patients with HFpEF [51], the most sensitive patient population regarding available treatment options. Even more, its effectiveness was also assessed in terms of other CV conditions, including MI, cardiac arrhythmias, and recently in cardiac dysfunction related to cancer therapy (Fig. 1). The summary of main clinical studies with S/V in different clinical settings is presented in Table 1 (Ref. [8, 52, 53, 54, 55, 56, 57]).

**Table 1.** **Main clinical studies with S/V in different clinical settings**.

Trial, design	Study population (n), inclusion criteria	Intervention, median follow-up	Primary outcomes
PARADIGM-HF [8] multicenter, randomized, double-blind study	HFrEF patients (n = 8442)	S/V 200 mg BID or enalapril 10 mg BID	20% reduction in composite of CV mortality and HF hospitalization with S/V
	EF ≤35%, NYHA II-IV, increased NT-proBNP levels	27 months	
TRANSITION [52] multicenter, randomized, open-label, parallel-group study	HFrEF patients with ADHF (n = 1002)	S/V 200 mg BID	Around 50 % of HFrEF patients stabilized after ADHF achieved target dose of S/V
	EF ≤35%	10 weeks	
PIONEER-HF [53] multicenter, randomized, double-blind study	HFrEF patients with ADHF (n = 736)	S/V 200 mg BID or enalapril 10 mg BID	Significant reduction of NT-proBNP levels with S/V
	EF ≤35%, NYHA II-IV	2 months	
PROVE-HF [54] multicenter, open-label, single-arm study	HFrEF patients (n = 794)	S/V 200 mg BID	Decrease in NT-proBNP levels correlated with improved echocardiographic markers of cardiac volume and function
	EF ≤35%, stable patients	12 months	
PARAGON-HF [55] multicenter, randomized, double-blind, parallel group study	HFpEF (n = 4822)	S/V 200 mg BID or valsartan 160 mg BID	No significant difference in composite endpoint of total HF hospitalization and CV mortality between S/V versus valsartan
	EF ≥45%, NYHA II–IV, increased NT-proBNP levels, structural heart disease	35 months	
PARALLAX [56] multicenter, randomized, double-blind study	HFpEF (n = 2572)	S/V 200 mg BID or background medication (enalapril, valsartan, or placebo)	Significant reduction of NT-proBNP levels at 12 weeks, but no significant difference in 6MWT at 24 weeks
	EF >40%, increased NT-proBNP levels, reduced life quality, structural heart disease	6 months	
PARADISE-MI [57] multicenter, randomized, double-blind study, parallel-group study	post-MI (n = 4650)	S/V 200 mg BID or ramipril 5 mg BID	No significant difference in composite of CV mortality or incident HF between S/V and ramipril
	EF ≤40% and/or pulmonary congestion, and at least 1 from 8 risk factors	23 months	

Abbreviations: BID, twice a day; ADHF, acute decompensated heart failure.

### 4.1 S/V in HFrEF

The landmark PARADIGM-HF trial was the largest HF trial ever, enrolling more than 8 thousand ambulatory patients with HFrEF (New York Heart Association (NYHA) class II–IV, EF ≤35%). During 27-months of a median follow-up, S/V reduced primary outcomes (CV death and HF hospitalization) by 20% when compared with ACEi (enalapril) [8]. This exceptional superiority of S/V over ACEi subsequently changed both American and European guidelines for HFrEF treatment [5, 9]. According to ESC guidelines, S/V was recommended instead of ACEi/ARB in patients with HFrEF (EF ≤35%), who remained symptomatic despite optimal treatment (including ACEi, BB, and MRA) [5]. The benefits of S/V observed in PARADIGM-HF were further confirmed in real-world studies. One retrospective cohort, involving a total of 132 patients with HFrEF (NYHA II–III), reported that in addition to BB and MRA, S/V reduced the risks from mortality and HF hospitalization at 6 months, when compared with standard therapy [58].

Since the PARADIGM-HF trial has been released, numerous sub-analyses have been performed concerning various aspects of S/V treatment regarding ACEi. While HF may occur due to various etiologies (ischemic or non-ischemic), S/V seems to be equally effective in diverse HFrEF population enrolled in PARADIGM-HF, irrespectively of specific pathophysiological state responsible for its development [59]. Furthermore, S/V is proved to be superior over enalapril in preventing the HFrEF clinical progression in surviving patients [60]. Moreover, in comparison to enalapril, an early benefit of S/V was also observed in regard to reduction of 30-day readmissions due to any cause and HF (by 26% and 38%, respectively) after discharge from HF hospitalization [61]. One observational study reported that S/V improved exercise capacity assessed by a 6-minute walk test (6MWT) in stable, symptomatic HFrEF patients [62]. Furthermore, in the open-label, PARASAIL study, HF patients (majority with NYHA II class) treated with S/V had improved mean 6-minute walking distance at 6-month follow-up, as well the quality of life [63]. However, there is limited data on S/V effects in patients with advanced HFrEF (NYHA class IV), while the PARADIGM-HF trial involved around 1% of patients in this NYHA category at baseline [8]. However, not so promising came the recent results from double-blind, randomized LIFE trial which demonstrated that in comparison to valsartan, S/V was not superior in reducing NT-proBNP levels nor in improving clinical outcomes (number of days alive, out of hospital, and free from HF events) in comorbid HFrEF patients (NYHA class IV) [64]. On the other hand, early initiation of S/V seems feasible in patients HFrEF confronted with recent acute decompensation either in a hospital setting or early after discharge, as reported in the TRANSITION and PIONEER-HF studies [52, 53]. The ESC Expert Consensus 2019 guidelines and the 2019 ACC Expert Consensus Decision Pathway for Hospitalized Patients reported that this agent might be considered in hospitalized patients with new-onset HFrEF or decompensation of chronic HF [65, 66].

As a common feature in HFrEF, cardiac dilatation is a predictor of poor outcome, while its reversal improves patients’ prognosis [67]. Thus, reverse cardiac remodeling is a significant marker of effective treatment and a predictor of better prognosis for HFrEF patients [68]. In the PARADIGM-HF trial, the risk of CV death or HF hospitalization increased by 10% for each 5% drop in LVEF, suggesting LVEF as an important outcome predictor [69]. One retrospective cohort by Almufleh and colleagues [70], reported that S/V improved LVEF (an increase from 25% to 33%) and reversed cardiac remodeling after 3-months of follow up compared to standard treatment. Similarly, Bayard and colleagues [71] observed that 3-month treatment with S/V significantly improved several echocardiographic parameters in patients with HFrEF, including LVEF (+3.6% in absolute value). Interestingly, more significant LV improvement has been observed in those with lower LV dilatation at baseline, suggesting that S/V could provide more prominent effects on LV when administered at the early HFrEF stage [71]. Most recently, a study by Paolini and colleagues [72] reported that during a 2-year follow-up, S/V treatment led to reverse cardiac remodeling in more than half of enrolled HFrEF patients, which usually occurred within 12 months after drug initiation. The authors also suggested that early ARNI implementation could prevent or postpone the ICD implantation in these patients.

The absence of a decrease in NT-proBNP levels in patients treated for HF is thought to be associated with poorer LV function and size [73]. In the PROVE-HF (Prospective Study of Biomarkers, Symptom Improvement and Ventricular Remodeling During Entresto Therapy for Heart Failure Study) trial, a decrease in circulating levels of NT-proBNP correlated with improved markers of cardiac function and volume in patients with HFrEF treated with S/V, suggesting reverse cardiac remodelling as its possible mechanism of benefit [54].

Among adverse events observed in PARADIGM-HF, only hypotension was significantly more common in patients treated with S/V than in those assigned to enalapril [8]. However, more patients who discontinued their study medication due to adverse events were receiving enalapril, while no difference in rates of hypotension-related therapy discontinuation was observed between these two groups. As for the other adverse events observed in PARADIGM-HF, mild angioedema was more common in patients treated with S/V, but without significant difference regarding those receiving enalapril. Interestingly, fewer patients treated with S/V experienced cough, elevated serum potassium, and elevated serum creatinine levels in comparison to those in the enalapril group [8].

In the PARADIGM-HF trial, the target dose of S/V was 97/103 mg twice a day, while the target dose of enalapril was 10 mg twice a day. However, almost half of the patients in both groups required a dose reduction, which was associated with a higher risk of major CV events in comparison to those who maintained on target doses [74]. Although, patients intolerant to maximal S/V doses, who were therefore prescribed with lower S/V doses still had more benefit than those receiving lower enalapril doses [74]. Nevertheless, there is still a large discrepancy between clinical trials and real-world studies in terms of initiation, titration, and adherence to S/V treatment. One study reported slow adoption of S/V in the real setting in the first 18 months after its FDA approval for HFrEF management, suggesting high cost as an important limiting factor for drug initiation and continuation [75]. The results from the CHAMP-HF Registry pointed out that 27% of HFrEF patients were not prescribed with an adequate guideline-directed treatment (ACEi/ARB or S/V) regardless of the absence of contraindications [76]. Furthermore, only 13% of HFrEF patients were using S/V, while only 14% were on maximal target doses [76]. Results from a large retrospective cohort conducted in Germany showed that two-thirds of patients treated with S/V were initially prescribed with the lowest dose, while up-titration was attempted in less than 50% of patients during the following 6 months [77]. The authors pointed out that it is necessary to identify the barriers responsible for missing S/V up-titration, as well as the importance of raising awareness among physicians on this matter [77]. The real-world data from Taiwan also showed that HFrEF patients were prescribed with lower doses than those included in the PARADIGM-HF trial [78]. There are various reasons for S/V underutilization, such as high cost, clinical factors (including older age, low BP, renal impairment, etc.), or even physician-related therapeutic inertia or fear from side effects [79]. Although clinical studies should not be a strict instruction for the use of certain drugs, but rather a guide to an individualized approach to each patient [80], raising awareness about timely implementation and adequate dose titration of guideline-directed medical treatment should be of particular interest, considering the observed benefits. In the future, randomized controlled trials are needed to determine the optimal dose of S/V [80].

Another important fact that needs to be addressed is that PARADIGM-HF trial had two phases of run-in period (enalapril active run-in period and LCZ696 active run-in period) with different duration of time, in whom the patients who tolerated both medications were randomized into the trial [81], which could suggest that individuals particularly susceptible to the hypotension were excluded from the study before randomization [82]. This part of the study design is of great importance, while run-in periods limit the benefits to a specific group of patients who are hard to be recognized in the clinical practice [83]. Furthermore, in PARADIGM-HF, patients assigned to S/V received valsartan in maximal dose in comparison to those on half-maximal enalapril dose, which can also be a reason for debate the origin of observed effects [83].

It seems that HFrEF patients would have particular benefit from both S/V and an SGLT2i [79], since these drug classes have a different mechanisms of action in patients with HF and their cardioprotective benefits are independent of each other [84]. Cardioprotection observed with SGLT2i is suggested to be regardless of neuro-hormonal antagonism [84] and it is most probably achieved with natriuretic/diuretic effect of these agents, but also other systemic, hemodynamic, and direct cardiac effects seem to have important involvement [85].

According to the latest ESC guidelines, in the absence of contraindications or intolerance, a cornerstone treatment for HFrEF patients includes ACEi/ARNI, BB, and MRA, up-titrated if possible to the target doses in clinical trials or maximally tolerated doses. In addition to optimal pharmacological treatment, SGLT2i (empagliflozin or dapagliflozin) are also recommended to HFrEF patients to reduce the risk of CV death and HF mortality, irrespectively from the presence of diabetes [4]. The present evidence suggests that HFrEF patients would have the most benefit from early initiation of the 4-drug treatment strategy (including ARNI, BB, MRA, and SGLT2i) in regard to death, HF hospitalization, and symptoms reduction [86]. A recent ACC consensus reported that the first-line therapy in patients with new-onset of symptomatic HFrEF should include ACEi/ARB/ARNI and BB, which need to be timely up-titrated to target or maximally tolerated doses [10]. In addition, adding an MRA and/or SGLT2i should be performed carefully in regard to estimated glomerular filtration rate and plasma potassium levels [10]. Furthermore, Packer and McMurray proposed a novel three-step strategy for the initiation of 4 drugs in euvolemic HFrEF patients during 4 weeks [87]. The first step would include concomitant initiation of BB and SGLT2i, the second step involves the initiation of ARNI, and the third step the initiation of MRA, with the possibility of an individualized approach if necessary [87]. The authors stated that this kind of therapeutic strategy would speed up the initiation of all 4 medications and improve their efficacy and tolerability in HFrEF patients [87].

In addition, another novelty in HFrEF treatment is FDA approval of vericiguat, an oral soluble guanylyl cyclase activator, for reducing the risk of CV mortality and HF hospitalization in patients with HFrEF worsening [88]. One network meta-analysis involving major trials with S/V, SGLT2i, and vericiguat, reported that in HFrEF patients these three drug classes had similar effects on outcomes, with only dapagliflozin being superior against vericiguat in HF hospitalization risk [89]. Interestingly, both S/V and vericiguat share a similar signaling pathway, cGMP-PKG pathway, and therefore may induce more prominent hypotensive effect which could protect from resistant hypertension (HTN) and HF development [90]. However, future investigations need to provide more detailed research regarding interaction between these two agents.

### 4.2 S/V in HFpEF

Many patients with HF suffer from HFpEF, which accounts for 22–73% of total HF cases, depending on the used definition [5]. It is associated with similarly poor survival rates as HFrEF [91]. HFpEF is more frequent in women and the elderly, with HTN, DM, and obesity being among common risk factors in this population of patients [92]. Despite the armamentarium of therapeutics proven effective for treating HFrEF, previous studies on HFpEF haven’t succeeded to develop an agent powerful enough to reduce morbidity and mortality in these patients [93].

The PARAMOUNT-HF trial, a phase II trial designed to assess the effects of ARNI against ARB in HFpEF patients, revealed promising results. In 301 HFpEF patients (NYHA II–IV) enrolled in this study, S/V was superior to valsartan in reducing the NT-proBNP levels at 12-weeks, and in lowering left atrial size and improving NYHA class at 36-weeks of treatment [51]. Moreover, BP reductions were also more significant at both 12 and 36-weeks of treatment in a group of patients receiving S/V compared to those assigned to valsartan [51]. A further sub-analysis of the PARAMOUNT-HF trial demonstrated a decrease in high sensitive Troponin I (hsTnI) levels in HFpEF patients treated with ARNI in regard to ARB, suggesting that S/V has the potential to ameliorate myocardial injury in HFpEF patients [94]. These long-awaited results have shed the light on HFpEF management and encouraged further research on S/V in this population of patients.

The PARAGON-HF trial, which enrolled more than 4 thousand patients with HFpEF (EF ≥45%), was the following study determined to investigate the effect of S/V on hard outcomes (CV death and total—first and recurrent—HF hospitalization). S/V showed no significant benefit over valsartan in reducing composite outcome of total HF hospitalizations and CV death [55]. However, the effectiveness of this agent is reported to be more prominent in women with HFpEF and higher LVEF compared to men, which implied the need for sex-based ranges when considering its benefits based on the EF measurements [95]. Results from the PARALLAX study, enrolling 2572 HFpEF patients, showed that S/V significantly reduced the levels of NT-proBNP compared to individualized medical therapy, however in terms of health status and exercise capacity, no significant difference has been observed [56].

There could be several explanations for different outcomes of S/V treatment efficacy in HFrEF and HFpEF patients. Large inconsistency in therapeutic response between HFrEF and HFpEF may actually originate from different pathophysiological drivers in these two entities or even in phenotypic diversity across the HFpEF spectrum [96]. Furthermore, the active comparator to S/V was enalapril in PARADIGM-HF, while in PARAGON-HF it was valsartan, which could have a potential influence on observed outcomes [8, 55]. In their study, Solomon and colleagues [97] performed a pooled analysis of combined data from both trials in order to access the effectiveness of S/V across the EF spectrum. The authors pointed out that the efficacy of S/V varies by EF values, with the most prominent benefits seen in patients with EF below normal (mid-range or borderline EF). These observations could be due to later therapy initiation in patients with higher EF or prevalence of cardiac stiffness in those with EF >50% leading to the lower effectiveness of S/V [79].

Cardiac fibrosis is undoubtedly recognized as one of the significant pathophysiological drivers in HFpEF, which occurs independently of its etiology [98]. Therefore its targeting could probably ensure significant cardioprotection. In a study by Zile and colleagues [99], S/V decreased profibrotic biomarkers in patients with HFrEF enrolled in PARADIGM-HF. Moreover, Cunningham and colleagues [100] provided further valuable evidence on antifibrotic features of S/V in the setting of HFpEF. As it was shown, HFpEF patients enrolled in the PARAGON-HF trial had increased biomarkers of extracellular matrix (ECM) dysregulation, which were associated with the risk of further HF events in this population. At the same time, S/V favorably affected these biomarkers revealing its antifibrotic potential [100].

In addition, there is an ongoing, randomized, double-blinded PARAGLIDE study investigating the effects of S/V versus valsartan alone on NT-proBNP values, as well as clinical outcomes, safety, and tolerability in HFpEF patients (EF >40%) with acute decompensated HF [101]. Hopefully, this study will provide us with novel insights into possible S/V use in the HFpEF population of patients.

### 4.3 S/V in MI Patients 

MI remains the most common cause of HF, which may develop due to diverse pathophysiological mechanisms depending on the time of its occurrence [102]. Although several studies confirmed lower rate of HF in post-MI patients with implementation of primary percutaneous coronary interventions, only a few had a longer follow-up period [102]. After demonstrating the beneficial effects of S/V in a diverse population of patients with HFrEF, further investigations were dedicated to determining its impact in patients following acute MI (AMI). Moreover, in the RECOVER-LV trial, which involved 93 patients with LV systolic dysfunction late after MI, S/V has shown no superiority over valsartan regarding reverse cardiac remodeling effects [103]. On the other hand, the data from the most recent meta-analysis involving four studies pointed to the beneficial effects of early S/V treatment after acute MI reflected through improved LVEF and reduced MACE incidence in comparison to ACEi [104]. However, S/V showed no superiority in reducing the incidence of cardiac death, HF hospitalization, MI or adverse side effects [104]. The SAVE-STEMI trial compared the efficacy of S/V against ramipril in patients with ST-segment elevation MI [105]. While there was no significant difference between these two treatment strategies after 1 month, treatment with S/V for 6 months prove to be more effective in decreasing MACE, as well as in improving EF and LV remodeling in these patients [105]. Most recently, in a study by Chen and colleagues [106], combined treatment with S/V and bisoprolol seem to be more effective in improving cardiac function and lowering the rate of adverse events during cardiac rehabilitation of patients with AMI and left-sided HF after PCI in comparison to bisoprolol monotherapy.

Finally, the most recent evidence emerged from the PARADISE-MI, the first large trial which compared the efficacy of ARNI versus ACEi in post-MI patients with LV dysfunction (EF ≤40%) and/or pulmonary congestion with at least one of eight additional risk-augmenting factors [57]. This study included a total of 5661 post-MI patients, who were randomized to receive S/V or ramipril within 12 h to one week after index AMI. None of the patients had a medical history of prior HF. Unfortunately, during a 2-year follow-up, S/V failed to significantly reduce the rate of CV death, HF hospitalization, or outpatient HF requiring treatment in patients following AMI compared to ramipril [57]. However, more research in this area could provide us with additional insights into the potential use of S/V in MI patients. Assessing the effects of S/V on larger or specific population of MI patients as well as in combination with other guideline-approved medications for MI seem to be of particular importance, while it could potentially reveal specific subgroup of patients expiriencing significant benefits of it usage in this clinical setting.

### 4.4 S/V in Rhythm Disturbances

Sudden cardiac death (SCD) represents the major cause of death in HF patients [107]. As the most robust parameter related to SCD, reduced LVEF is considered as an indication for implantable cardioverter-defibrillator (ICD) implantation for primary prevention of SCD [108]. Importantly, HFrEF patients assigned to S/V lived up to 2 years longer with less possibility to die from SCD or HF worsening than those prescribed with enalapril [109, 110]. The exact mechanism responsible for reduced SCD in HFrEF patients treated with S/V is not entirely clarified, while there are conflicting results in the present literature regarding its antiarrhythmic potential [108, 111, 112, 113, 114, 115].

In a study by Vincent and colleagues [112], which involved 108 of patients prescribed with S/V six patients presented with ventricular arrhythmic storm early after S/V initiation, which required drug discontinuation. Paradoxically, one observational study involving 167 patients with dilated cardiomyopathy (ischemic/non-ischemic etiology) and dual-chamber ICD reported that treatment with S/V reduced the incidence of atrial and ventricular arrhythmias and improved ICD electrical atrial parameters during 12-month follow up [116]. Similarly, de Diego and colleagues [117] reported a lower rate of non-sustained ventricular tachycardia and premature ventricular contraction (PVC) in HFrEF patients treated with S/V than those prescribed with ACE/ARB. Furthermore, a correlation between plasma NT-proBNP levels and hourly PVC rate was observed and decreased with S/V treatment [117]. In a study by Martens and colleagues [118], HFrEF patients with implanted ICD or cardiac resynchronization therapy (CRT) receiving S/V had a lower rate of SCD, which could be at least partially driven by the reduced onset of ventricular tachyarrhythmia’s. Reverse cardiac remodelling observed during treatment with S/V treatment could be one of the contributing mechanisms responsible for reduced risk of arrhythmias.

One retrospective cohort reported increased peak atrial longitudinal strain (PALS) in patients with HF (NYHA II or II–III) with a history of atrial fibrillation (AF) prescribed with S/V [119], as well as reduced rate of AF episodes in comparison to standard therapies, during 12-months follow up. While PALS is considered a marker of reservoir function of atrial chambers, its augmentation under S/V treatment emphasize the beneficial effects of this agent on atrial filling and thus more pronounced LV ejection during systole [119]. Furthermore, same group of authors reported that at ventricular level S/V increases both LVEF and global longitudinal strain in HFrEF patients [120], with possible ability for reverse remodeling of atrias and regulation of heart rhythm [121]. Beneficial effects of S/V in the setting of AF may be explained by its favourable influence on electroanatomic atrial remodelling, and it should be one particular field of examination in future studies.

### 4.5 S/V in Chemotherapy-induced LV Dysfunction

In previous years, significant progress has been made in the treatment of patients with malignant diseases. However, along with improved survival in these patients, prolonged exposure to cancer therapies led to an increased risk of their adverse effects [122]. Cancer therapy-related cardiac dysfunction (CTRCD) is considered in case of reduced EF of more than 10% from baseline to an EF <53% [123]. In cancer survivors, ACEi/ARBs and BB are recommended therapeutics for symptomatic HF or asymptomatic cardiac dysfunction, as well as for preventing further deterioration of LV function (when a drop of EF >10% below reference is registered) [124]. However, HF with LV dysfunction still largely contributes to morbidity and mortality burden in patients with malignant diseases [125].

Although the PARADIGM-HF trial demonstrated beneficial effects of S/V in patients with HFrEF of diverse etiology, the evidence on its efficacy and safety in patients with CTRCD is lacking. Initially, potential cardioprotective effects of S/V were reported in a few case reports and case-series studies involving patients with malignant diseases [126, 127, 128, 129]. Most recently, in a study by Gregorietti and colleagues [130], authors reported potential benefit from S/V in the population of patients with breast cancer and cardiac dysfunction, reflected through improved LVEF, LV diameters, and diastolic dysfunction, as well as symptoms and 6MWT parameters. Furthermore, one multicentric retrospective study involving 67 CTRCD patients treated with S/V revealed promising results [131]. The use of S/V was associated with reverse cardiac remodelling (improved LVEF and LV volumes) along with improved exercise tolerance and reductions in NT-proBNP levels [131]. Moreover, tolerability of S/V was reported as good, with only a few patients (6%) experiencing an adverse event [131]. Similarly, significant improvement in LV volumes and LVEF assessed by cardiac magnetic resonance, along with reductions in NT-pro-BNP levels were observed in CTRCD patients treated with S/V [132]. Although these studies involved small number of patients, promising observations should encourage further investigations to assess more robust conclusions on the use of S/V in cancer survivors with CTRCD.

## 5. Cardioprotection of S/V and its Proposed Mechanisms—Evidence from Animal Studies

Multiple preclinical studies showed the protective effects of S/V on cardiac function in the setting of various CV pathologies. It is well established that NEP inhibition may influence the circulating levels of peptides other than NPs, which may additionally contribute to favourable effects of S/V in the setting of different CV pathologies. Therefore, various animal studies tried to unravel mechanistic aspects of S/V contributing to its cardioprotection (Table 2, Ref. [133, 134, 135, 136, 137, 138, 139, 140, 141, 142, 143, 144, 145, 146, 147, 148, 149, 150, 151, 152, 153, 154]). In the next section, we summarized the current preclinical knowledge on potential mechanisms of S/V in CVD modelling.

**Table 2.** **Summary of selected preclinical studies with S/V in cardiac disorders**.

Animal model	Dose/duration	Main molecular mechanism(s) and effects	Cardiac and hemodynamic effects	Ref.
post-MI (LAD ligation) in SD rats	68 mg/kg daily, 4 weeks	Reduction of fibrosis rate in peri-infarct and remote myocardium	Reduction of HW, hypertrophy and fibrosis, LV remodeling, LVEDd; improvement of LV function and EF	[133]
MHD in C57BL/6J mice	100 mg/kg daily, 16 weeks	Reduction of fibrosis, hypertrophy, and collagen production in heart; reduction of myocardial oxidative stress	Reduction of total wall thickness, LV mass, LVEDP, E/Em; improvement of E/A ratio, Em, RPP	[134]
ISO-exposed Wistar rats	60 mg/kg daily, 1 week	Reduction of cardiac interstitial fibrosis and expression of TGF-β1, Col1a1, Ccl2	Reduction of serum NT-proBNP, SBP; attenuation of the LVEDP and Dp/dt max increase	[135]
MI (LAD-ligation) in SD rats	68 mg/kg daily, 4 weeks	Inhibition of myocardial fibroblast proliferation and collagen synthesis through downregulation of TGF-β1/Smads signaling	Reduction of LVEDd, LVEDs, IVSd, LVPWd; increase of EF, FS	[136]
HFrEF in diabetic C57BL/6J mice	60 mg/kg daily, 4 weeks	Reduction of LV fibrosis; decreased expression of TGF-β, ANP	Reduction of serum NT-proBNP, HW/BW; improvement of EF, SV, CO, SW	[137]
HFpEF in Dahl/SS rats	68 mg/kg daily, 4 weeks	Inhibition of cardiac fibrosis by suppressing the TGF-β1/Smad3 signaling pathway	Reduction of serum NT-proBNP, SBP, LV/BW, (Wet lung-Dry lung)/BW, LA/BW, IVSd, LVPWd, LA; correction of LV mass, E/A and E/E′; improvement of EF, FS, LV DD	[138]
DOX-exposed Wistar rats	68 mg/kg daily, 4–6 weeks	Altered extracellular matrix remodeling secondary to a reduction in myocardial MMP activity	Preservation of EF and FS	[139]
HFpEF in ZSF1 obese rats	60 mg/kg daily, 12 weeks	Reduction of perivascular fibrosis, decrease of Collagen I and III, ANP and BNP expressions, decrease of MMP-2 activity, increase of cGMP levels and phosphor-titin levels	Reduction of serum NT-proBNP, HW, LVESP, LVEDP, and LV stiffness, MAP in aorta, RV volume capacity; improvement of DD, EF and endothelial-dependent vasodilation in carotid arteries	[140]
Debanding surgery in C57BL/6 J mice with aortic banding	60 mg/kg daily, 4 weeks	Inhibition of NF-κB-mediated NLRP3 inflammasome activation	Reduction of HW/BW, LV mass, LVESd; improvement of EF, FS	[141]
MI (LAD ligation) in C57BL/6J mice	20 mg/kg daily, 4 weeks	Suppression of pro-inflammatory cytokines and ECM degradation by macrophages	Reduction of rate of death due to LV rupture, LVEDd, LVESd, plasma aldosterone, aldosterone/cGMP ratio; increase of survival rate, FS, plasma cGMP levels	[142]
TAC in C57Bl6/J mice	57 mg/kg twice daily, 4 weeks	Inhibition of Rho signaling via stabilization of ANF-induced PKG signaling	Reduction of SBP, LV mass, systolic and diastolic internal dimensions and volumes; improvement of EF, FS; preservation of E/E’ values	[143]
Post MI (LAD ligation) in SD rats	60 mg/kg daily, 4 weeks	Reduction of cardiomyocyte hypertrophy, cardiac fibrosis and collagen I expression in the non-infarct and border zone, expressions of ANP, βMHC, and TIMP2	Reduction of HW, LV weight, LA weight, lung weight, LV filling pressures, LVESV, LVEDP, LVPW thickness, LV diastolic wall strain, LV compliance; improvement of EF, FS, ESPV relationship; preservation of dP/dt max and dP/dt min normalized to LVEDV	[144]
TAC in C57BL/6 mice	60 mg/kg daily, 4 weeks	Inhibition of inflammatory response in blood and heart tissues, reduction of cardiac fibrosis and hypertrophy, improvement of ventricular remodeling	Reduction of LVEDs, LVEDd, LVPWs, LVPWd, IVSs, IVSd, LV mass; improvement of EF, FS	[145]
Post-TAC in C57BL/6 mice	60 mg/kg daily, 4 weeks	Reduction of cardiac fibrosis and preservation of cardiomyocyte morphology	Reduction of LA, EF, IVSd, LVPWd, LVEDd, LVESd	[146]
AF rabbit model	10 mg/kg twice daily, 3 weeks	Attenuation of atrial electrical and structural remodeling probably via calcineurin/NFAT pathway, preservation of cardiomyocyte morphology	Reduction of serum NT-proBNP, AF incidence; preservation of rapid pacing-induced atria and RV enlargement	[147]
diabetic CMP in C57BL/6 mice	60 mg/kg daily 16 weeks	Inhibition the HG- or diabetes-induced JNK/p38MAPK phosphorylation and NF-kB nuclear translocation; decrease of apoptosis, oxidative stress, fibrosis, collagen I, and collagen III levels	Reduction of serum NT-proBNP; improvement of LV contractility and diastolic function	[148]
MI (LAD ligation) in SD rats	68 mg/kg daily, 1 week	Inhibition of TAK1/JNK signalling cascade; reduction of interstitial fibrosis, collagen volume fraction, serum levels of inflammatory factors (IL‑1 β and IL‑18) and ROS accumulation and downregulate the expression levels of NLRP3; downregulation of pro-caspase-l, pro-IL-lβ and pro-IL-18 expression	Reduction of myocardial injury and improved ventricular remodeling	[149]
EAM in BALB/c mice	20 mg/kg daily, 2 weeks	Inhibition of Th17 cell differentiation (independent from the NLRP3 inflammasome pathway); Reduction of inflammatory markers	Reduction of HW/BW, pathological scores of heart sections and cTnT	[150]
MCT-induced PH and Hypoxia-induced PH in SD rats	68 mg/kg daily, 2 weeks	Increase of ANP and CNP; Restoration of the down-regulated NPRs protein expression, preservation of cGMP content of lung tissues; decrease of IL-1β, IL-6, and TNF-α concentrations in blood	Reduction of MAP, mPAP, PVR, RV weight to LV + S weight ratio, pulmonary artery wall thickness, fully muscularization of pulmonary arterioles and improved non-muscular arterioles	[151]
DOX-induced dilatative CMP in Balb/c mice	60 mg/kg daily, 4 weeks	Preservation of mitochondrial function via reduced activity of fission protein Drp1; reduction of myocardial hypertrophy, fibrosis and cell size, apoptosis and cardiomyocyte contractile dysfunction	Reduction of serum NT-proBNP, HW/BW; improvement in HW/TL, EF, LVEDd, LVESd	[152]
TAC-induced pressure overload HF In C57BL/6	20 mg/kg daily, 4 weeks	Anti-hypertrophic effect by ameliorating oxidative stress via the Sirt3/MnSOD pathway; reduction of cardiac hypertrophy, fibrosis, ANP, BNP, β-MHC, myocardial ROS and myocardial apoptosis	Reduction of HW/BW, LW/BW, HW/TL; improvement of EF, FS and hypertrophy contractile dysfunction	[153]
HF by I/R injury (LAD ligation) in SHR	68 mg/kg daily, 4 or 6 weeks	Reduction of extension of infarct border zone, collagen volume fraction, collagen I and collagen III expressions, TIMP2, TGF-β; improved endothelium-independent vascular reactivity and vascular compliance; increased circulating plasma and myocardial NO levels and PKG protein levels	Reduction of serum NT-proBNP, LVEDd, LVESd, LVEDP; improvement of EF	[154]

Abbreviations: AF, atrial fibrillation; ANP, atrial natriuretic peptide; BNP, brain natriuretic peptide; BW, body weight; Ccl2, C motif chemokine ligand 2; cGMP, cyclic guanosine monophosphate; CMP, cardiomyopathy; CNP, C-type natriuretic peptide; CO, cardiac output; Col1a1, collagen type 1 alpha 1; cTnT, cardiac Troponin T; DBP, diastolic blood pressure; DD, diastolic dysfunction; DOX, doxorubicin; EAM, experimental autoimmune myocarditis; ECM, extracellular matrix; EF, ejection fraction; ESPV, end-systolic pressure volume relationship; FS, fractional shortening; HFpEF, heart failure with preserved ejection fraction; HFrEF, heart failure with reduced ejection fraction; HW, heart weight; HW/TL, heart weight/tibial length; IL, interleukin; ISO, isoproterenol; IVSd, interventricular septum thickness at the end of diastole; IVST, intraventricular septum thickness; LA, left atrial internal dimensions; LAD, left anterior descending artery; LV, left ventricle; LVEDd, left ventricle end-diastolic diameter; LVEDP, left ventricle end-diastolic pressure; LVEDV, left ventricle end-diastolic volume; LVESd, left ventricle end-systolic diameter; LVESP, left ventricle end-systolic pressure; LVESV, left ventricle end-systolic volume; LVPWd, left ventricular posterior wall thickness at the end of diastole; LVPWs, left ventricular posterior wall thickness at the end of systole; LVSP, left ventricle systolic pressure; MAP, mean arterial pressure; MCT, monocrotaline; βMHC, β myosin heavy chain; MI, myocardial infarction; MMP, matrix metalloproteinase; mPAP, mean pulmonary arterial pressure; NT-proBNP, N-terminal pro b-type natriuretic peptide; PH, pulmonary hypertension; PVR, pulmonary vascular resistance; ref., references; RPP, heart rate x developed pressure; RV, right ventricle; SBP, systolic blood pressure; SD, Sprague Dawley; SV, stroke volume; SW, stroke work; TAC, transverse aortic constriction; TIMP, tissue inhibitor of metalloproteinases; TGF-β, transforming growth factor-beta; TNF-α, tumour necrosis factor-α.

### 5.1 Antifibrotic and Antihypertrophic Effects of S/V

Cardiac fibrosis as a frequent companion of heart diseases, leads to dilatation, cardiomyocyte hypertrophy, and apoptosis, with HF as an ultimate pathophysiological event [155]. Accumulation of activated myofibroblast at the injury site stands for a significant driver of the fibrotic process in cardiac tissue [98]. In recent years, antifibrotic properties of S/V have been investigated in various preclinical studies involving majorly MI and HF modelling. Early investigations in this field were reported in a study by von Lueder and colleagues [133], where chronic S/V treatment, initiated one week after MI induction in rats, preserved cardiac function and remodelling by reducing myocardial hypertrophy and fibrosis in peri-infarcted and non-infarcted remote myocardium. Similar findings were reported in a study by Kusaka and colleagues [156], where, comparing to valsartan, treatment with S/V inhibited cardiac fibrosis and hypertrophy in rats with metabolic syndrome and HTN. Furthermore, Croteau and colleagues [134] demonstrated that S/V was superior over valsartan in improving diastolic function and reducing cardiac interstitial fibrosis in obesity-related metabolic heart disease in mice. In a study by Miyoshi and colleagues [135], authors reported decreased cardiac fibrosis along with reduced mRNA expressions of transforming growth factor (TGF)-β1 and Collagen Type I Alpha 1 (Col1α1) in isoproterenol-induced cardiac damage in rats, in comparison to valsartan monotherapy. However, no significant decrease in cardiac hypertrophy has been observed [135].

Present literature evidence supports the role of TGF-β and Smad signalling cross-talk in the development of cardiac fibrosis [157]. Therefore, its suppression could be beneficial in profibrotic cardiovascular conditions. It is well described that Smad2 and Smad3 transcription factors, as a part of the TGF signalling pathway, become phosphorylated after TGF-β activation, form a heteromeric complex with Smad4, translocate to the nucleus to control gene expression involved in fibrotic processes [136]. In a study by Suematsu and colleagues [137], S/V attenuated LV fibrosis by suppressing the mRNA expression of TGF-β regarding ARB treatment in streptozotocin-induced diabetic mice with HFrEF. Furthermore, treatment with S/V suppressed the TGF-β/Smads signalling pathway in both MI and HFpEF rat model [136, 138], suggesting that antifibrotic S/V features are manifest in various CV pathologies in animals.

Matrix metalloproteinases (MMPs) are members of a zinc-dependent enzyme family responsible for degrading specific molecules that constitute extracellular matrix (ECM). Various studies implied the role of MMPs overexpression in the setting of LV remodeling and myocardial dysfunction, while inhibiting MMPs activity may be a promising therapeutic approach for preventing HF [158]. In a study by Boutagy and colleagues [139], cardioprotective effects of chronic S/V treatment were at least partially mediated by reduced activity of cardiac MMPs in doxorubicin-induced cardiotoxicity. Increased activity of MMP-2 by 25% was observed in the HFpEF rat model compared to controls, while 12-week treatment with S/V attenuated its activity by around 30% [140]. Similarly, a decrease in α-SMA-positive cells along with reductions of profibrotic markers (TGF-β, Col I, and MMP-2) have been reported after S/V treatment in pressure unloaded mice [141]. Furthermore, Ishii and colleagues [142] demonstrated that treatment with S/V decreased MMP-9 mRNA expression, which was related to a lower rate of cardiac rupture after acute MI in rats compared to enalapril treatment.

In their research, Burke and colleagues [143] reported that in the pressure-overload HF model in mice, S/V exerted antifibrotic effects by directly affecting cardiac myofibroblast via PKG-dependent inhibition of RhoA, which is involved in myofibroblast transition and activation. In a study by Kompa and colleagues [144], 4-week treatment with S/V decreased cardiac hypertrophy and fibrosis to a similar extent as perindopril alone. At the same time, gene expressions of ANP and βMHC, as well as TIMP2, were markedly reduced with S/V versus perindopril. However, MMP9 was not specifically altered with MI induction in comparison to Sham animals, neither did the applied therapies result in significant MMP9 changes. Furthermore, Ge and colleagues [145] reported that reduced cardiac fibrosis, hypertrophy, and lymphatic remodelling in mice with TAC treated with S/V was driven by its inhibitory effect on inflammatory response.

Suo and colleagues’ [146] study showed that S/V treatment was superior to valsartan in preserving left atrial and left atrial appendage remodelling and reduced atrial fibrosis in the pressure-overload mice model. Beneficial effects of S/V treatment were also observed in the AF rabbit model, where this agent preserved atrial structural remodelling by reducing cardiac fibrosis [147]. Furthermore, in rats with induced pulmonary hypertension (PH), 3-week treatment with S/V prevented RV remodelling by its favourable effects on RV pressure, hypertrophy, the orientation of collagen and myofiber as well as tissue stiffening [159].

### 5.2 Antiinflammatory Effects of S/V

Up to date, there is little evidence supporting the anti-inflammatory potential of S/V on its observed cardioprotection in clinical studies [160]. However, according to the previous basic researches, treatment with S/V decreased the circulating levels of proinflammatory cytokines, such as MMP-8, IL-6, and monocyte chemoattractant protein (MCP)-1 in ApoE2/2 mice in regard to valsartan [161]. Similarly, treatment with S/V reduced proinflammatory mediators in the blood and hearts of TAC mice [145]. In a study by Ge and colleagues [148], S/V alleviated diabetic cardiomyopathy in mice, which was an effect partially driven by its ability to inhibit inflammation. Furthermore, S/V was more effective than enalapril in improving survival by inhibiting inflammatory response in post-AMI setting in mice [142].

It is well known that Nucleotide-Binding Domain-Like Receptor Protein 3 (NLRP3) inflammasome signalling, primarily involved in inducing secretion of proinflammatory cytokines, contributes to the pathophysiological cascade of various chronic diseases such as HF [162]. On the other hand, it was previously proposed that the TGF‑β-activated kinase 1 (TAK1)/JNK pathway has an important role in regulating the activation of the NLRP3 inflammasome [163]. In a study by Shen and colleagues [149], S/V decreased myocardial injury and LV remodelling and inhibited the expression of the NLRP3 inflammasome in MI rats through suppression of the TAK1/JNK signalling pathway. In another study, the beneficial effects of S/V treatment in pressure unloaded mice were at least partially driven by modulation of NF-κB-dependent inhibition of NLRP3 inflammasome activation [141]. On the other hand, Liang and colleagues [150] reported the beneficial effects of S/V treatment regarding expressing acute inflammation phase mediators in the myocarditis mice model. The authors suggested that sGC/NF-κB p65 signalling pathway was involved in the observed inhibition of Th17 cell differentiation, independently of the NLRP3 inflammasome activation [150]. Moreover, protective effects of S/V has been observed in both monocrotaline- and hypoxia-induced PH in rats. The most probable mechanisms involved RAAS inhibition, promotion of ANP/NPR-A/cGMP and CNP/NPR-B/cGMP pathway, restoration of the NPR-C signalling, as well as the anti-inflammatory effects [151].

### 5.3 Antiapoptotic Effects of S/V

Cardiac apoptosis has an important role in various CVDs, including MI and HF [164]. Several preclinical studies highlighted the antiapoptotic effects of S/V as potential mediators of improved cardiac structure and function. In a study by Xia and colleagues [152], 4-week treatment with S/V improved DOX-induced cardiomyopathy in adult mice. In this report, cardioprotective effects of S/V are thought to be (at least partially) driven by its ability to reduce the activity of fission protein dynamin-related protein 1 (Drp1) involved in the apoptotic cascade and thus preserve mitochondrial function [152]. In a study by Peng and colleagues [153], pressure overload HF was induced by a transverse aortic constriction in mice, which were further treated with S/V. Authors reported that a 4-week treatment with S/V decreased myocardial apoptosis assessed by TUNEL staining, along with significantly reduced expressions of apoptotic markers (Bax and Bcl-2) [153]. Similarly, diabetic cardiomyopathy mice administered with S/V had attenuated protein expression of apoptotic markers (cleaved caspase-3 and Bax/Bcl-2 ratio), along with improvement in cardiac dysfunction and remodelling [148]. S/V treatment also tended to reduce myocardial apoptotic rate in pressure unloaded mice, although these effects did not reach a significant level [141].

### 5.4 S/V and Oxidative Stress

Oxidative stress, as an imbalance between ROS production and antioxidative defense capacity [165], is suggested as one of the contributors in the pathophysiological cascade of various heart conditions, including HF [166]. In ISO-induced MI in rats, combined administration of sacubitril and valsartan in high dose preserved myocardial tissue damage and reduced infarcted area [167]. The authors further reported that the potential cardioprotective mechanism of S/V involves its ability to reduce oxidative stress [167]. Furthermore, Croteau and colleagues [134] reported that S/V decreased oxidative stress in the myocardium more prominently than valsartan, an effect mirrored through reductions of oxidized lipid 4-Hydroxy-2-nonenal (4-HNE) in mice with metabolic heart disease.

Oxidative stress can activate nuclear factor (NF)-κB, a transcriptional factor which is known to be involved in the expression of proinflammatory cytokines and apoptosis-related genes [168], therefore therapeutic targeting this complex network between NF-κB and oxidative stress may be particularly important in diseased states [169]. In a study by Ge and colleagues [148], treatment with S/V improved ventricular remodeling and dysfunction in a rodent model of diabetic cardiomyopathy, partially attributed to its ability to inhibit oxidative damage by inhibiting JNK/p38MAPK phosphorylation and NF-kB nuclear translocation. Furthermore, S/V diminished myocardial oxidative stress in TAC-induced HF in mice via Sirt3/MnSOD pathway [153]. On the other hand, it is well documented that oxidative stress may influence vascular tone by inducing endothelial dysfunction majorly by decreasing nitric oxide (NO) bioavailability [170]. In their recent paper, Trivedi and colleagues [154] demonstrated that in comparison to valsartan, 4-week treatment with S/V enhanced NO bioavailability and improved vascular function in spontaneously hypertensive rats with HF.

## 6. Future Perspectives and Conclusions

Given the adopted knowledge on S/V cardioprotection, it would be of great interest to further investigate its effectiveness and safety in different CV pathologies. Moreover, evidence on simultaneous administration of S/V and other existing and developing CV therapeutics could provide novel insights into possible synergistic benefits. Bearing in mind its antiinflamatory potential, it would be important to conduct more preclinical studies concerning the efficacy of S/V in the setting of inflammatory heart diseases, including myocarditis, while this agent may improve both cardiac dysfunction and inflammatory response in this clinical setting. Of note, discovering other signalling pathways affected by S/V should be of particular interest for basic researches, while it can provide additional understanding of its cardioprotective mechanisms.

## References

[1] Roth GA, Mensah GA, Johnson CO, Addolorato G, Ammirati E, Baddour LM (2020). Global burden of cardiovascular diseases and risk factors, 1990–2019: update from the GBD 2019 study. *Journal of the American College of Cardiology*.

[2] Mishra P, Samanta L (2012). Oxidative stress and heart failure in altered thyroid States. *The Scientific World Journal*.

[3] James SL, Abate D, Abate KH, Abay SM, Abbafati C, Abbasi N (2018). Global, regional, and national incidence, prevalence, and years lived with disability for 354 diseases and injuries for 195 countries and territories, 1990–2017: a systematic analysis for the Global Burden of Disease Study 2017. *The Lancet*.

[4] McDonagh TA, Metra M, Adamo M, Gardner RS, Baumbach A, Böhm M (2021). 2021 ESC Guidelines for the diagnosis and treatment of acute and chronic heart failure. *European Heart Journal*.

[5] Ponikowski P, Voors AA, Anker SD, Bueno H, Cleland JG, Coats AJ (2016). 2016 ESC Guidelines for the diagnosis and treatment of acute and chronic heart failure: The Task Force for the diagnosis and treatment of acute and chronic heart failure of the European Society of Cardiology (ESC). Developed with the special contribution of the Heart Failure Association (HFA) of the ESC. *European Journal of Heart Failure*.

[6] Volpe M, Carnovali M, Mastromarino V (2016). The natriuretic peptides system in the pathophysiology of heart failure: from molecular basis to treatment. *Clinical Science*.

[7] Oktay AA, Rich JD, Shah SJ (2013). The emerging epidemic of heart failure with preserved ejection fraction. *Current Heart Failure Reports*.

[8] McMurray JJV, Packer M, Desai AS, Gong J, Lefkowitz MP, Rizkala AR (2014). Angiotensin-neprilysin inhibition versus enalapril in heart failure. *New England Journal of Medicine*.

[9] Yancy CW, Jessup M, Bozkurt B, Butler J, Casey DE, Colvin MM (2017). 2017 ACC/AHA/HFSA focused update of the 2013 ACCF/AHA guideline for the management of heart failure: a report of the American College of Cardiology/American Heart Association Task Force on Clinical Practice Guidelines and the Heart Failure Society of America. *Circulation*.

[10] Maddox TM, Januzzi JL, Allen LA, Breathett K, Butler J, Davis LL (2021). 2021 Update to the 2017 ACC Expert Consensus Decision Pathway for Optimization of Heart Failure Treatment: Answers to 10 Pivotal Issues About Heart Failure With Reduced Ejection Fraction: A Report of the American College of Cardiology Solution Set Oversight Committee. *Journal of the American College of Cardiology*.

[11] McMurray JJV, Solomon SD, Inzucchi SE, Køber L, Kosiborod MN, Martinez FA (2019). Dapagliflozin in patients with heart failure and reduced ejection fraction. *New England Journal of Medicine*.

[12] Packer M, Anker SD, Butler J, Filippatos G, Pocock SJ, Carson P (2020). Cardiovascular and renal outcomes with empaglifozin in heart failure. *New England Journal of Medicine*.

[13] Solomon SD, Jhund PS, Claggett BL, Dewan P, Køber L, Kosiborod MN (2020). Effect of Dapagliflozin in Patients with HFrEF Treated with Sacubitril/Valsartan: The DAPA-HF Trial. *JACC: Heart Failure*.

[14] Packer M, Anker SD, Butler J, Filippatos G, Ferreira JP, Pocock SJ (2021). Influence of neprilysin inhibition on the efficacy and safety of empagliflozin in patients with chronic heart failure and a reduced ejection fraction: the EMPEROR-Reduced trial. *European Heart Journal*.

[15] Levin ER, Gardner DG, Samson WK (1998). Natriuretic peptides. *New England Journal of Medicine*.

[16] de Bold AJ, Borenstein HB, Veress AT, Sonnenberg H (1981). A rapid and potent natriuretic response to intravenous injection of atrial myocardial extract in rats. *Life Sciences*.

[17] Currie MG, Geller DM, Cole BR, Siegel NR, Fok KF, Adams SP (1984). Purification and sequence analysis of bioactive atrial peptides (atriopeptins). *Science*.

[18] Kangawa K, Matsuo H (1984). Purification and complete amino acid sequence of alpha-human atrial natriuretic polypeptide (alpha-hANP). *Biochemical and Biophysical Research Communications*.

[19] Sudoh T, Kangawa K, Minamino N, Matsuo H (1988). A new natriuretic peptide in porcine brain. *Nature*.

[20] Mukoyama M, Nakao K, Hosoda K, Suga S, Saito Y, Ogawa Y (1991). Brain natriuretic peptide as a novel cardiac hormone in humans. Evidence for an exquisite dual natriuretic peptide system, atrial natriuretic peptide and brain natriuretic peptide. *Journal of Clinical Investigation*.

[21] Mukoyama M, Nakao K, Saito Y, Ogawa Y, Hosoda K, Suga S (1990). Increased human brain natriuretic peptide in congestive heart failure. *New England Journal of Medicine*.

[22] Sudoh T, Minamino N, Kangawa K, Matsuo H (1990). C-Type natriuretic peptide (CNP): a new member of natriuretic peptide family identified in porcine brain. *Biochemical and Biophysical Research Communications*.

[23] D’Elia E, Iacovoni A, Vaduganathan M, Lorini FL, Perlini S, Senni M (2017). Neprilysin inhibition in heart failure: mechanisms and substrates beyond modulating natriuretic peptides. *European Journal of Heart Failure*.

[24] Ogawa T, de Bold AJ (2014). The heart as an endocrine organ. *Endocrine Connections*.

[25] Rubattu S, Sciarretta S, Valenti V, Stanzione R, Volpe M (2008). Natriuretic peptides: an update on bioactivity, potential therapeutic use, and implication in cardiovascular diseases. *American Journal of Hypertension*.

[26] Potter LR, Abbey-Hosch S, Dickey DM (2006). Natriuretic peptides, their receptors, and cyclic guanosine monophosphate-dependent signaling functions. *Endocrine Reviews*.

[27] Nakagawa Y, Nishikimi T, Kuwahara K (2018). Atrial and brain natriuretic peptides: Hormones secreted from the heart. *Peptides*.

[28] Nussenzveig DR, Lewicki JA, Maack T (1990). Cellular mechanisms of the clearance function of type C receptors of atrial natriuretic factor. *Journal of Biological Chemistry*.

[29] Koh GY, Nussenzveig DR, Okolicany J, Price DA, Maack T (1992). Dynamics of atrial natriuretic factor-guanylate cyclase receptors and receptor-ligand complexes in cultured glomerular mesangial and renomedullary interstitial cells. *Journal of Biological Chemistry*.

[30] Seferovic JP, Solomon SD, Seely EW (2020). Potential mechanisms of beneficial effect of sacubitril/valsartan on glycemic control. *Therapeutic Advances in Endocrinology and Metabolism*.

[31] Potter LR (2011). Natriuretic peptide metabolism, clearance and degradation. *FEBS Journal*.

[32] Munagala VK, Burnett JC, Redfield MM (2004). The natriuretic peptides in cardiovascular medicine. *Current Problems in Cardiology*.

[33] Silver MA (2006). The natriuretic peptide system: kidney and cardiovascular effects. *Current Opinion in Nephrology and Hypertension*.

[34] Díez J (2017). Chronic heart failure as a state of reduced effectiveness of the natriuretic peptide system: implications for therapy. *European Journal of Heart Failure*.

[35] Young JB, Abraham WT, Stevenson LW, Horton DP, Elkayam U, Bourge RC (2002). Intravenous nesiritide vs nitroglycerin for treatment of decompensated congestive heart failure: a randomized controlled trial. *Journal of the American Medical Association*.

[36] O’Connor CM, Starling RC, Hernandez AF, Armstrong PW, Dickstein K, Hasselblad V (2011). Effect of nesiritide in patients with acute decompensated heart failure. *New England Journal of Medicine*.

[37] Sato N, Kajimoto K, Asai K, Mizuno M, Minami Y, Nagashima M (2010). Acute decompensated heart failure syndromes (ATTEND) registry. A prospective observational multicenter cohort study: rationale, design, and preliminary data. *American Heart Journal*.

[38] Tanaka TD, Sawano M, Ramani R, Friedman M, Kohsaka S (2018). Acute heart failure management in the USA and Japan: overview of practice patterns and review of evidence. *ESC Heart Failure*.

[39] Nagai T, Iwakami N, Nakai M, Nishimura K, Sumita Y, Mizuno A (2019). Effect of intravenous carperitide versus nitrates as first-line vasodilators on in-hospital outcomes in hospitalized patients with acute heart failure: Insight from a nationwide claim-based database. *International Journal of Cardiology*.

[40] Roques BP, Fournié-Zaluski MC, Soroca E, Lecomte JM, Malfroy B, Llorens C (1980). The enkephalinase inhibitor thiorphan shows antinociceptive activity in mice. *Nature*.

[41] Gros C, Souque A, Schwartz JC, Duchier J, Cournot A, Baumer P (1989). Protection of atrial natriuretic factor against degradation: diuretic and natriuretic responses after *in vivo* inhibition of enkephalinase (EC 3.4.24.11) by acetorphan. *Proceedings of the National Academy of Sciences of the United States of America*.

[42] Northridge DB, Jardine AG, Alabaster CT, Barclay PL, Connell JM, Dargie HJ (1989). Effects of UK 69 578: a novel atriopeptidase inhibitor. *The Lancet*.

[43] Bevan EG, Connell JMC, Doyle J, Carmichael HA, Davies DL, Lorimer AR (1992). Candoxatril, a neutral endopeptidase inhibitor: efficacy and tolerability in essential hypertension. *Journal of Hypertension*.

[44] Swedberg K, Held P, Kjekshus J, Rasmussen K, Rydén L, Wedel H (1987). Effects of enalapril on mortality in severe congestive heart failure. Results of the Cooperative North Scandinavian Enalapril Survival Study (CONSENSUS). *New England Journal of Medicine*.

[45] Yusuf S, Pitt B, Davis CE, Hood WB, Cohn JN (1991). Effect of enalapril on survival in patients with reduced left ventricular ejection fractions and congestive heart failure. *New England Journal of Medicine*.

[46] Packer M, Califf RM, Konstam MA, Krum H, McMurray JJ, Rouleau J (2002). Comparison of omapatrilat and enalapril in patients with chronic heart failure: the Omapatrilat Versus Enalapril Randomized Trial of Utility in Reducing Events (OVERTURE). *Circulation*.

[47] Dargad RR, Prajapati MR, Dargad RR, Parekh JD (2018). Sacubitril/valsartan: a novel angiotensin receptor-neprilysin inhibitor. *Indian Heart Journal*.

[48] Vardeny O, Miller R, Solomon SD (2014). Combined neprilysin and renin-angiotensin system inhibition for the treatment of heart failure. *JACC: Heart Failure*.

[49] Baxter GF (2004). The natriuretic peptides. *Basic Research in Cardiology*.

[50] Gu J, Noe A, Chandra P, Al-Fayoumi S, Ligueros-Saylan M, Sarangapani R (2010). Pharmacokinetics and pharmacodynamics of LCZ696, a novel dual-acting angiotensin receptor-neprilysin inhibitor (ARNi). *Journal of Clinical Pharmacology*.

[51] Solomon SD, Zile M, Pieske B, Voors A, Shah A, Kraigher-Krainer E (2012). The angiotensin receptor neprilysin inhibitor LCZ696 in heart failure with preserved ejection fraction: a phase 2 double-blind randomised controlled trial. *The Lancet*.

[52] Wachter R, Senni M, Belohlavek J, Straburzynska-Migaj E, Witte KK, Kobalava Z (2019). Initiation of sacubitril/valsartan in haemodynamically stabilised heart failure patients in hospital or early after discharge: primary results of the randomised TRANSITION study. *European Journal of Heart Failure*.

[53] Velazquez EJ, Morrow DA, DeVore AD, Duffy CI, Ambrosy AP, McCague K (2019). Angiotensin-Neprilysin Inhibition in Acute Decompensated Heart Failure. *New England Journal of Medicine*.

[54] Januzzi JL, Prescott MF, Butler J, Felker GM, Maisel AS, McCague K (2019). Association of Change in N-Terminal Pro–B-Type Natriuretic Peptide Following Initiation of Sacubitril-Valsartan Treatment with Cardiac Structure and Function in Patients with Heart Failure with Reduced Ejection Fraction. *Journal of the American Medical Association*.

[55] Solomon SD, McMurray JJV, Anand IS, Ge J, Lam CSP, Maggioni AP (2019). Angiotensin-Neprilysin Inhibition in Heart Failure with Preserved Ejection Fraction. *New England Journal of Medicine*.

[56] Pieske B, Wachter R, Shah SJ, Baldridge A, Szeczoedy P, Ibram G (2021). PARALLAX Investigators and Committee members. Effect of Sacubitril/Valsartan vs Standard Medical Therapies on Plasma NT-proBNP Concentration and Submaximal Exercise Capacity in Patients With Heart Failure and Preserved Ejection Fraction: The PARALLAX Randomized Clinical Trial. *Journal of the American Medical Association*.

[57] Pfeffer MA, Claggett B, Lewis EF, Granger CB, Køber L, Maggioni AP (2021). Angiotensin Receptor-Neprilysin Inhibition in Acute Myocardial Infarction. *New England Journal of Medicine*.

[58] De Vecchis R, Ariano C, Di Biase G, Noutsias M (2019). Sacubitril/valsartan for heart failure with reduced left ventricular ejection fraction : a retrospective cohort study. *Herz*.

[59] Simpson J, Jhund P, Rouleau J, Swedberg K, Zile M, Lefkowitz M (2017). Effect of sacubitril/valsartan compared with enalapril, according to etiology in PARADIGM-HF. *American College of Cardiology Scientific Sessions*.

[60] Packer M, McMurray JJ, Desai AS, Gong J, Lefkowitz MP, Rizkala AR (2014). Angiotensin Receptor Neprilysin Inhibition Cmpared With Enalapril on the Risk of Clinical Progression in Surviving Patients With Heart Failure. *Circulation*.

[61] Desai AS, Claggett BL, Packer M, Zile MR, Rouleau JL, Swedberg K (2016). Influence of Sacubitril/Valsartan (LCZ696) on 30-Day Readmission after Heart Failure Hospitalization. *Journal of the American College of Cardiology*.

[62] Beltrán P, Palau P, Domínguez E, Faraudo M, Núñez E, Guri O (2018). Sacubitril/valsartan and short-term changes in the 6-minute walk test: a pilot study. *International Journal of Cardiology*.

[63] Haddad H, Bergeron S, Ignaszewski A, Searles G, Rochdi D, Dhage P (2020). Canadian Real-World Experience of Using Sacubitril/Valsartan in Patients with Heart Failure with Reduced Ejection Fraction: Insight from the PARASAIL Study. *CJC Open*.

[64] Mann DL, Givertz MM, Vader JM, Starling RC, Shah P, McNulty SE (2022). Effect of Treatment With Sacubitril/Valsartan in Patients With Advanced Heart Failure and Reduced Ejection Fraction: A Randomized Clinical Trial. *JAMA Cardiology*.

[65] Seferovic PM, Ponikowski P, Anker SD, Bauersachs J, Chioncel O, Cleland JGF (2019). Clinical practice update on heart failure 2019: pharmacotherapy, procedures, devices and patient management. An expert consensus meeting report of the Heart Failure Association of the European Society of Cardiology. *European Journal of Heart Failure*.

[66] Hollenberg SM, Warner Stevenson L, Ahmad T, Amin VJ, Bozkurt B, Butler J (2019). 2019 ACC expert consensus decision pathway on risk assessment, management, and clinical trajectory of patients hospitalized with heart failure: a report of the American College of Cardiology Solution Set Oversight Committee. *Journal of the American College of Cardiology*.

[67] Reis Filho JR, Cardoso JN, Cardoso CM, Pereira-Barretto AC (2015). Reverse Cardiac Remodeling: A Marker of Better Prognosis in Heart Failure. *Arquivos Brasileiros De Cardiologia*.

[68] Konstam MA, Kramer DG, Patel AR, Maron MS, Udelson JE (2011). Left ventricular remodeling in heart failure: current concepts in clinical significance and assessment. *JACC: Cardiovascular Imaging*.

[69] Solomon SD, Claggett B, Desai AS, Packer M, Zile M, Swedberg K (2016). Influence of Ejection Fraction on Outcomes and Efficacy of Sacubitril/Valsartan (LCZ696) in Heart Failure with Reduced Ejection Fraction: the Prospective Comparison of ARNI with ACEI to Determine Impact on Global Mortality and Morbidity in Heart Failure (PARADIGM-HF) Trial. *Circulation: Heart Failure*.

[70] Almufleh A, Marbach J, Chih S, Stadnick E, Davies R, Liu P (2017). Ejection fraction improvement and reverse remodeling achieved with sacubitril/valsartan in heart failure with reduced ejection fraction patients. *American Journal of Cardiovascular Disease*.

[71] Bayard G, Da Costa A, Pierrard R, Roméyer-Bouchard C, Guichard JB, Isaaz K (2019). Impact of sacubitril/valsartan on echo parameters in heart failure patients with reduced ejection fraction a prospective evaluation. *IJC Heart and Vasculature*.

[72] Paolini C, Mugnai G, Dalla Valle C, Volpiana A, Ferraglia A, Frigo AC (2021). Effects and clinical implications of sacubitril/valsartan on left ventricular reverse remodeling in patients affected by chronic heart failure: a 24-month follow-up. *IJC Heart and Vasculature*.

[73] Gaggin HK, Truong QA, Rehman SU, Mohammed AA, Bhardwaj A, Parks KA (2013). Characterization and Prediction of Natriuretic Peptide “Nonresponse” during Heart Failure Management: Results from the ProBNP Outpatient Tailored Chronic Heart Failure (PROTECT) and the NT-proBNP-Assisted Treatment to Lessen Serial Cardiac Readmissions and Death (BATTLESCARRED) study. *Congestive Heart Failure*.

[74] Vardeny O, Claggett B, Packer M, Zile MR, Rouleau J, Swedberg K (2016). Efficacy of sacubitril/valsartan vs. enalapril at lower than target doses in heart failure with reduced ejection fraction: the PARADIGM-HF trial. *European Journal of Heart Failure*.

[75] Sangaralingham LR, Sangaralingham SJ, Shah ND, Yao X, Dunlay SM (2018). Adoption of Sacubitril/Valsartan for the Management of Patients with Heart Failure. *Circulation: Heart Failure*.

[76] Greene SJ, Butler J, Albert NM, DeVore AD, Sharma PP, Duffy CI (2018). Medical therapy for heart failure with reduced ejection fraction: The CHAMP-HF registry. *Journal of the American College of Cardiology*.

[77] Wachter R, Fonseca AF, Balas B, Kap E, Engelhard J, Schlienger R (2019). Real‐world treatment patterns of sacubitril/valsartan: a longitudinal cohort study in Germany. *European Journal of Heart Failure*.

[78] Hsiao FC, Wang CL, Chang PC, Lu YY, Huang CY, Chu PH (2020). Angiotensin Receptor Neprilysin Inhibitor for Patients with Heart Failure and Reduced Ejection Fraction: Real-World Experience from Taiwan. *Journal of Cardiovascular Pharmacology and Therapeutics*.

[79] Volpe M, Bauersachs J, Bayés-Genís A, Butler J, Cohen-Solal A, Gallo G (2021). Sacubitril/valsartan for the management of heart failure: a perspective viewpoint on current evidence. *International Journal of Cardiology*.

[80] Cleland JG, Pellicori P (2016). PARADIGM-HF: does dose matter. *European Journal of Heart Failure*.

[81] McMurray JJ, Packer M, Desai AS, Gong J, Lefkowitz MP, Rizkala AR (2013). Dual angiotensin receptor and neprilysin inhibition as an alternative to angiotensin-converting enzyme inhibition in patients with chronic systolic heart failure: rationale for and design of the Prospective comparison of ARNI with ACEI to Determine Impact on Global Mortality and morbidity in Heart Failure trial (PARADIGM-HF). *European Journal of Heart Failure*.

[82] Vardeny O, Claggett B, Kachadourian J, Pearson SM, Desai AS, Packer M (2018). Incidence, Predictors, and Outcomes Associated with Hypotensive Episodes among Heart Failure Patients Receiving Sacubitril/Valsartan or Enalapril: the PARADIGM-HF Trial (Prospective Comparison of Angiotensin Receptor Neprilysin Inhibitor with Angiotensin-Converting Enzyme Inhibitor to Determine Impact on Global Mortality and Morbidity in Heart Failure). *Circulation: Heart Failure*.

[83] Ahn R, Prasad V (2018). Do Limitations in the Design of PARADIGM-HF Justify the Slow Real World Uptake of Sacubitril/Valsartan (Entresto). *Cardiovascular Drugs and Therapy*.

[84] Fucili A, Cimaglia P, Severi P, Giannini F, Boccadoro A, Micillo M (2021). Looking for a Tailored Therapy for Heart Failure: Are We Capable of Treating the Patient Instead of the Disease. *Journal of Clinical Medicine*.

[85] Nikolic M, Zivkovic V, Jovic JJ, Sretenovic J, Davidovic G, Simovic S (2021). SGLT2 inhibitors: a focus on cardiac benefits and potential mechanisms. *Heart Failure Reviews*.

[86] Bauersachs J (2021). Heart failure drug treatment: the fantastic four. *European Heart Journal*.

[87] Packer M, McMurray JJV (2021). Rapid evidence‐based sequencing of foundational drugs for heart failure and a reduced ejection fraction. *European Journal of Heart Failure*.

[88] Merck Announces U.S (2021). FDA Approval of VERQUVO® (vericiguat). News release. Merck. https://www.merck.com/news/merck-announces-u-s-fda-approval-of-verquvo-vericiguat/.

[89] Aimo A, Pateras K, Stamatelopoulos K, Bayes-Genis A, Lombardi CM, Passino C (2021). Relative Efficacy of Sacubitril-Valsartan, Vericiguat, and SGLT2 Inhibitors in Heart Failure with Reduced Ejection Fraction: a Systematic Review and Network Meta-Analysis. *Cardiovascular Drugs and Therapy*.

[90] Gallo G, Rubattu S, Volpe M (2021). Targeting Cyclic Guanylate Monophosphate in Resistant Hypertension and Heart Failure: are Sacubitril/Valsartan and Vericiguat Synergistic and Effective in both Conditions. *High Blood Pressure and Cardiovascular Prevention*.

[91] Bhatia RS, Tu JV, Lee DS, Austin PC, Fang J, Haouzi A (2006). Outcome of heart failure with preserved ejection fraction in a population-based study. *New England Journal of Medicine*.

[92] Lam CS, Donal E, Kraigher-Krainer E, Vasan RS (2011). Epidemiology and clinical course of heart failure with preserved ejection fraction. *European Journal of Heart Failure*.

[93] Clark KAA, Velazquez EJ (2020). Heart Failure with Preserved Ejection Fraction. *Journal of the American Medical Association*.

[94] Jhund PS, Claggett BL, Voors AA, Zile MR, Packer M, Pieske BM (2014). Elevation in High-Sensitivity Troponin T in Heart Failure and Preserved Ejection Fraction and Influence of Treatment with the Angiotensin Receptor Neprilysin Inhibitor LCZ696. *Circulation: Heart Failure*.

[95] McMurray JJV, Jackson AM, Lam CSP, Redfield MM, Anand IS, Ge J (2020). Effects of Sacubitril-Valsartan Versus Valsartan in Women Compared With Men With Heart Failure and Preserved Ejection Fraction: Insights From PARAGON-HF. *Circulation*.

[96] Shah SJ, Kitzman DW, Borlaug BA, van Heerebeek L, Zile MR, Kass DA (2016). Phenotype-Specific Treatment of Heart Failure with Preserved Ejection Fraction: A Multiorgan Roadmap. *Circulation*.

[97] Solomon SD, Vaduganathan M, L Claggett B, Packer M, Zile M, Swedberg K (2020). Sacubitril/valsartan across the spectrum of ejection fraction in heart failure. *Circulation*.

[98] Sweeney M, Corden B, Cook SA (2020). Targeting cardiac fibrosis in heart failure with preserved ejection fraction: mirage or miracle. *EMBO Molecular Medicine*.

[99] Zile MR, O’Meara E, Claggett B, Prescott MF, Solomon SD, Swedberg K (2019). Effects of Sacubitril/Valsartan on Biomarkers of Extracellular Matrix Regulation in Patients with HFrEF. *Journal of the American College of Cardiology*.

[100] Cunningham JW, Claggett BL, O’Meara E, Prescott MF, Pfeffer MA, Shah SJ (2020). Effect of Sacubitril/Valsartan on Biomarkers of Extracellular Matrix Regulation in Patients with HFpEF. *Journal of the American College of Cardiology*.

[101] (2022). United States National Library of Medicine Clinical Trials Registry. https://clinicaltrials.gov/ct2/show/record/NCT03988634.

[102] Cahill TJ, Kharbanda RK (2017). Heart failure after myocardial infarction in the era of primary percutaneous coronary intervention: Mechanisms, incidence and identification of patients at risk. *World Journal of Cardiology*.

[103] Docherty KF, Campbell RT, Brooksbank KJM, Dreisbach JG, Forsyth P, Godeseth RL (2021). Effect of Neprilysin Inhibition on Left Ventricular Remodeling in Patients With Asymptomatic Left Ventricular Systolic Dysfunction Late After Myocardial Infarction. *Circulation*.

[104] Zhao J, Zeng Y, Shen X (2021). Efficacy and safety of early initiation of Sacubitril/Valsartan in patients after acute myocardial infarction: a meta‐analysis. *Clinical Cardiology*.

[105] Rezq A, Saad M, El Nozahi M (2021). Comparison of the Efficacy and Safety of Sacubitril/Valsartan versus Ramipril in Patients with ST-Segment Elevation Myocardial Infarction. *American Journal of Cardiology*.

[106] Chen C, Wu X, Li Y, Peng Y (2021). Study on the application effect of bisoprolol combined with sacubitril valsartan sodium tablets in the cardiac rehabilitation of patients with acute myocardial infarction combined with left heart failure after percutaneous coronary intervention (PCI). *Annals of Palliative Medicine*.

[107] Rosanio S, Schwarz ER, Vitarelli A, Zarraga IGE, Kunapuli S, Ware DL (2007). Sudden death prophylaxis in heart failure. *International Journal of Cardiology*.

[108] Grabowski M, Ozierański K, Balsam P, Dąbrowski R, Farkowski MM, Gackowski A (2019). The effect of sacubitril/valsartan on the occurrence of ventricular arrhythmia and the risk of sudden cardiac death in patients with chronic heart failure with reduced left ventricular ejection fraction. Expert opinion of the Heart Rhythm and Heart Failure Sections of the Polish Cardiac Society. *Kardiologia Polska*.

[109] Claggett B, Packer M, McMurray JJ, Swedberg K, Rouleau J, Zile MR (2015). Estimating the Long-Term Treatment Benefits of Sacubitril-Valsartan. *New England Journal of Medicine*.

[110] Desai AS, McMurray JJ, Packer M, Swedberg K, Rouleau JL, Chen F (2015). Effect of the angiotensin-receptor-neprilysin inhibitor LCZ696 compared with enalapril on mode of death in heart failure patients. *European Heart Journal*.

[111] El-Battrawy I, Pilsinger C, Liebe V, Lang S, Kuschyk J, Zhou X (2019). Impact of Sacubitril/Valsartan on the Long-Term Incidence of Ventricular Arrhythmias in Chronic Heart Failure Patients. *Journal of Clinical Medicine*.

[112] Vicent L, Juárez M, Martín I, García J, González-Saldívar H, Bruña V (2018). Ventricular Arrhythmic Storm after Initiating Sacubitril/Valsartan. *Cardiology*.

[113] Okutucu S, Oto A (2019). Electrical Storm after Initiating Sacubitril/Valsartan: Arrhythmic Paradox. *Cardiology*.

[114] Valentim Gonçalves A, Pereira-da-Silva T, Galrinho A, Rio P, Moura Branco L, Soares R (2020). Antiarrhythmic Effect of Sacubitril-Valsartan: Cause or Consequence of Clinical Improvement. *Journal of Clinical Medicine*.

[115] Sarrias A, Bayes-Genis A (2018). Is Sacubitril/Valsartan (also) an Antiarrhythmic Drug. *Circulation*.

[116] Russo V, Bottino R, Rago A, Papa AA, Liccardo B, Proietti R (2020). The Effect of Sacubitril/Valsartan on Device Detected Arrhythmias and Electrical Parameters among Dilated Cardiomyopathy Patients with Reduced Ejection Fraction and Implantable Cardioverter Defibrillator. *Journal of Clinical Medicine*.

[117] de Diego C, González-Torres L, Núñez JM, Centurión Inda R, Martin-Langerwerf DA, Sangio AD (2018). Effects of angiotensin-neprilysin inhibition compared to angiotensin inhibition on ventricular arrhythmias in reduced ejection fraction patients under continuous remote monitoring of implantable defibrillator devices. *Heart Rhythm*.

[118] Martens P, Nuyens D, Rivero-Ayerza M, Van Herendael H, Vercammen J, Ceyssens W (2019). Sacubitril/valsartan reduces ventricular arrhythmias in parallel with left ventricular reverse remodeling in heart failure with reduced ejection fraction. *Clinical Research in Cardiology*.

[119] De Vecchis R, Paccone A, Di Maio M (2020). Favorable Effects of Sacubitril/Valsartan on the Peak Atrial Longitudinal Strain in Patients with Chronic Heart Failure and a History of one or more Episodes of Atrial Fibrillation: a Retrospective Cohort Study. *Journal of Clinical Medicine Research*.

[120] De Vecchis R, Paccone A, Di Maio M (2019). Sacubitril/Valsartan Therapy for 14 Months Induces a Marked Improvement of Global Longitudinal Strain in Patients with Chronic Heart Failure: a Retrospective Cohort Study. *Cardiology Research*.

[121] De Vecchis R, Paccone A, Di Maio M (2020). Upstream Therapy for Atrial Fibrillation Prevention: the Role of Sacubitril/Valsartan. *Cardiology Research*.

[122] Schamroth Pravda N, Kornowski R (2018). Unmet Needs and Therapeutic Strategies in Cardio-Hemato-Oncology. *Acta Haematologica*.

[123] Plana JC, Galderisi M, Barac A, Ewer MS, Ky B, Scherrer-Crosbie M (2014). Expert consensus for multimodality imaging evaluation of adult patients during and after cancer therapy: a report from the American Society of Echocardiography and the European Association of Cardiovascular Imaging. *Journal of the American Society of Echocardiography*.

[124] Zamorano JL, Lancellotti P, Rodriguez Muñoz D, Aboyans V, Asteggiano R, Galderisi M (2016). 2016 ESC Position Paper on cancer treatments and cardiovascular toxicity developed under the auspices of the ESC Committee for Practice Guidelines. The Task Force for cancer treatments and cardiovascular toxicity of the European Society of Cardiology (ESC). *European Heart Journal*.

[125] Yoon HJ, Kim KH, Kim JY, Park HJ, Cho JY, Hong YJ (2016). Chemotherapy-Induced Left Ventricular Dysfunction in Patients with Breast Cancer. *Journal of Breast Cancer*.

[126] De Vecchis R, Paccone A (2020). A case series about the favorable effects of sacubitril/valsartan on anthracycline cardiomyopathy. *SAGE Open Medical Case Reports*.

[127] Sheppard CE, Anwar M (2019). The use of sacubitril/valsartan in anthracycline-induced cardiomyopathy: a mini case series. *Journal of Oncology Pharmacy Practice*.

[128] Morris K, Wagner S, Ravichandran A, Patel A, Chaudhry S, Garcia-Cortes R (2019). Novel use of valsartan-sacubitril as treatment for trastuzumab induced cardiomyopathy. *Journal of the American College of Cardiology*.

[129] Lupi A, Ariotti S, De Pace D, Ferrari I, Bertuol S, Monti L (2021). Sacubitril/Valsartan to Treat Heart Failure in a Patient with Relapsing Hairy Cell Leukaemia: Case Report. *Clinical Medicine Insights: Cardiology*.

[130] Gregorietti V, Fernandez TL, Costa D, Chahla EO, Daniele AJ (2020). Use of Sacubitril/valsartan in patients with cardio toxicity and heart failure due to chemotherapy. *Cardio-oncology*.

[131] Martín‐Garcia A, López‐Fernández T, Mitroi C, Chaparro‐Muñoz M, Moliner P, Martin‐Garcia AC (2020). Effectiveness of sacubitril-valsartan in cancer patients with heart failure. *ESC Heart Failure*.

[132] Martín-García A, Díaz-Peláez E, Martín-García AC, Sánchez-González J, Ibáñez B, Sánchez PL (2020). Myocardial function and structure improvement with sacubitril/valsartan in cancer therapy-induced cardiomyopathy. *Revista EspañOla De Cardiología*.

[133] von Lueder TG, Wang BH, Kompa AR, Huang L, Webb R, Jordaan P (2015). Angiotensin Receptor Neprilysin Inhibitor LCZ696 Attenuates Cardiac Remodeling and Dysfunction after Myocardial Infarction by Reducing Cardiac Fibrosis and Hypertrophy. *Circulation: Heart Failure*.

[134] Croteau D, Qin F, Chambers JM, Kallick E, Luptak I, Panagia M (2020). Differential Effects of Sacubitril/Valsartan on Diastolic Function in Mice with Obesity-Related Metabolic Heart Disease. *JACC: Basic to Translational Science*.

[135] Miyoshi T, Nakamura K, Miura D, Yoshida M, Saito Y, Akagi S (2019). Effect of LCZ696, a dual angiotensin receptor neprilysin inhibitor, on isoproterenol-induced cardiac hypertrophy, fibrosis, and hemodynamic change in rats. *Cardiology Journal*.

[136] Wu M, Guo Y, Wu Y, Xu K, Lin L (2021). Protective Effects of Sacubitril/Valsartan on Cardiac Fibrosis and Function in Rats With Experimental Myocardial Infarction Involves Inhibition of Collagen Synthesis by Myocardial Fibroblasts Through Downregulating TGF-β1/Smads Pathway. *Frontiers in Pharmacology*.

[137] Suematsu Y, Miura S, Goto M, Matsuo Y, Arimura T, Kuwano T (2016). LCZ696, an angiotensin receptor-neprilysin inhibitor, improves cardiac function with the attenuation of fibrosis in heart failure with reduced ejection fraction in streptozotocin-induced diabetic mice. *European Journal of Heart Failure*.

[138] Zhang W, Liu J, Fu Y, Ji H, Fang Z, Zhou W (2020). Sacubitril/Valsartan Reduces Fibrosis and Alleviates High-Salt Diet-Induced HFpEF in Rats. *Frontiers in Pharmacology*.

[139] Boutagy NE, Feher A, Pfau D, Liu Z, Guerrera NM, Freeburg LA (2020). Dual Angiotensin Receptor-Neprilysin Inhibition with Sacubitril/Valsartan Attenuates Systolic Dysfunction in Experimental Doxorubicin-Induced Cardiotoxicity. *JACC: CardioOncology*.

[140] Schauer A, Adams V, Augstein A, Jannasch A, Draskowski R, Kirchhoff V (2021). Sacubitril/Valsartan Improves Diastolic Function But Not Skeletal Muscle Function in a Rat Model of HFpEF. *International Journal of Molecular Sciences*.

[141] Li X, Zhu Q, Wang Q, Zhang Q, Zheng Y, Wang L (2020). Protection of Sacubitril/Valsartan against Pathological Cardiac Remodeling by Inhibiting the NLRP3 Inflammasome after Relief of Pressure Overload in Mice. *Cardiovascular Drugs and Therapy*.

[142] Ishii M, Kaikita K, Sato K, Sueta D, Fujisue K, Arima Y (2017). Cardioprotective Effects of LCZ696 (Sacubitril/Valsartan) after Experimental Acute Myocardial Infarction. *JACC: Basic to Translational Science*.

[143] Burke RM, Lighthouse JK, Mickelsen DM, Small EM (2019). Sacubitril/Valsartan Decreases Cardiac Fibrosis in Left Ventricle Pressure Overload by Restoring PKG Signaling in Cardiac Fibroblasts. *Circulation: Heart Failure*.

[144] Kompa AR, Lu J, Weller TJ, Kelly DJ, Krum H, von Lueder TG (2018). Angiotensin receptor neprilysin inhibition provides superior cardioprotection compared to angiotensin converting enzyme inhibition after experimental myocardial infarction. *International Journal of Cardiology*.

[145] Ge Q, Zhao L, Liu C, Ren X, Yu Y, Pan C (2020). LCZ696, an Angiotensin Receptor-Neprilysin Inhibitor, Improves Cardiac Hypertrophy and Fibrosis and Cardiac Lymphatic Remodeling in Transverse Aortic Constriction Model Mice. *BioMed Research International*.

[146] Suo Y, Yuan M, Li H, Zhang Y, Li Y, Fu H (2019). Sacubitril/Valsartan Improves Left Atrial and Left Atrial Appendage Function in Patients with Atrial Fibrillation and in Pressure Overload-Induced Mice. *Frontiers in Pharmacology*.

[147] Li LY, Lou Q, Liu GZ, Lv J, Yun F, Li T (2020). Sacubitril/valsartan attenuates atrial electrical and structural remodelling in a rabbit model of atrial fibrillation. *European Journal of Pharmacology*.

[148] Ge Q, Zhao L, Ren XM, Ye P, Hu ZY (2019). LCZ696, an angiotensin receptor-neprilysin inhibitor, ameliorates diabetic cardiomyopathy by inhibiting inflammation, oxidative stress and apoptosis. *Experimental Biology and Medicine*.

[149] Shen J, Fan Z, Sun G, Qi G (2021). Sacubitril/valsartan (LCZ696) reduces myocardial injury following myocardial infarction by inhibiting NLRP3‑induced pyroptosis via the TAK1/JNK signaling pathway. *Molecular Medicine Reports*.

[150] Liang W, Xie BK, Ding PW, Wang M, Yuan J, Cheng X (2021). Sacubitril/Valsartan Alleviates Experimental Autoimmune Myocarditis by Inhibiting Th17 Cell Differentiation Independently of the NLRP3 Inflammasome Pathway. *Frontiers in Pharmacology*.

[151] Liu S, Wang Y, Lu S, Hu J, Zeng X, Liu W (2021). Sacubitril/valsartan treatment relieved the progression of established pulmonary hypertension in rat model and its mechanism. *Life Sciences*.

[152] Xia Y, Chen Z, Chen A, Fu M, Dong Z, Hu K (2017). LCZ696 improves cardiac function via alleviating Drp1-mediated mitochondrial dysfunction in mice with doxorubicin-induced dilated cardiomyopathy. *Journal of Molecular and Cellular Cardiology*.

[153] Peng S, Lu XF, Qi YD, Li J, Xu J, Yuan T (2020). LCZ696 Ameliorates Oxidative Stress and Pressure Overload-Induced Pathological Cardiac Remodeling by Regulating the Sirt3/MnSOD Pathway. *Oxidative Medicine and Cellular Longevity*.

[154] Trivedi RK, Polhemus DJ, Li Z, Yoo D, Koiwaya H, Scarborough A (2018). Combined Angiotensin Receptor-Neprilysin Inhibitors Improve Cardiac and Vascular Function Via Increased NO Bioavailability in Heart Failure. *Journal of the American Heart Association*.

[155] Travers JG, Kamal FA, Robbins J, Yutzey KE, Blaxall BC (2016). Cardiac Fibrosis: the Fibroblast Awakens. *Circulation Research*.

[156] Kusaka H, Sueta D, Koibuchi N, Hasegawa Y, Nakagawa T, Lin B (2015). LCZ696, Angiotensin II Receptor-Neprilysin Inhibitor, Ameliorates High-Salt-Induced Hypertension and Cardiovascular Injury more than Valsartan alone. *American Journal of Hypertension*.

[157] Saadat S, Noureddini M, Mahjoubin-Tehran M, Nazemi S, Shojaie L, Aschner M (2021). Pivotal Role of TGF-β/Smad Signaling in Cardiac Fibrosis: Non-coding RNAs as Effectual Players. *Frontiers in Cardiovascular Medicine*.

[158] Lee RT (2001). Matrix metalloproteinase inhibition and the prevention of heart failure. *Trends in Cardiovascular Medicine*.

[159] Sharifi Kia D, Benza E, Bachman TN, Tushak C, Kim K, Simon MA (2020). Angiotensin Receptor‐Neprilysin Inhibition Attenuates Right Ventricular Remodeling in Pulmonary Hypertension. *Journal of the American Heart Association*.

[160] Valentim Goncalves A, Pereira-da-Silva T, Galrinho A, Rio P, Moura Branco L, Soares R, Ilhao Moreira R (2020). C-reactive protein reduction with sacubitril-valsartan treatment in heart failure patients. *American Journal of Cardiovascular Disease*.

[161] Zhang H, Liu G, Zhou W, Zhang W, Wang K, Zhang J (2019). Neprilysin Inhibitor-Angiotensin II Receptor Blocker Combination Therapy (Sacubitril/valsartan) Suppresses Atherosclerotic Plaque Formation and Inhibits Inflammation in Apolipoprotein E- Deficient Mice. *Scientific Reports*.

[162] Butts B, Gary RA, Dunbar SB, Butler J (2015). The Importance of NLRP3 Inflammasome in Heart Failure. *Journal of Cardiac Failure*.

[163] Okada M, Matsuzawa A, Yoshimura A, Ichijo H (2014). The Lysosome Rupture-activated TAK1-JNK Pathway Regulates NLRP3 Inflammasome Activation. *Journal of Biological Chemistry*.

[164] Kim NH, Kang PM (2010). Apoptosis in cardiovascular diseases: mechanism and clinical implications. *Korean Circulation Journal*.

[165] Rahal A, Kumar A, Singh V, Yadav B, Tiwari R, Chakraborty S (2014). Oxidative stress, prooxidants, and antioxidants: the interplay. *BioMed Research International*.

[166] Senoner T, Dichtl W (2019). Oxidative Stress in Cardiovascular Diseases: Still a Therapeutic Target. *Nutrients*.

[167] Imran M, Hassan MQ, Akhtar MS, Rahman O, Akhtar M, Najmi AK (2018). Sacubitril and valsartan protect from experimental myocardial infarction by ameliorating oxidative damage in Wistar rats. *Clinical and Experimental Hypertension*.

[168] Wu X, Luo A, Zhou Y, Ren J (2014). N-acetylcysteine reduces oxidative stress, nuclear factor‑κB activity and cardiomyocyte apoptosis in heart failure. *Molecular Medicine Reports*.

[169] Lingappan K (2018). NF-κB in Oxidative Stress. *Current Opinion in Toxicology*.

[170] Schulz E, Gori T, Münzel T (2011). Oxidative stress and endothelial dysfunction in hypertension. *Hypertens Res*.

